# The role of RNA modification in hepatocellular carcinoma

**DOI:** 10.3389/fphar.2022.984453

**Published:** 2022-09-02

**Authors:** Qiang Feng, Dongxu Wang, Tianyi Xue, Chao Lin, Yongjian Gao, Liqun Sun, Ye Jin, Dianfeng Liu

**Affiliations:** ^1^ Laboratory Animal Center, College of Animal Science, Jilin University, Changchun, China; ^2^ School of Grain Science and Technology, Jilin Business and Technology College, Changchun, China; ^3^ Department of Gastrointestinal Colorectal and Anal Surgery, China-Japan Union Hospital of Jilin University, Changchun, China; ^4^ Department of Pediatrics, First Hospital of Jilin University, Changchun, China; ^5^ School of Pharmacy, Changchun University of Chinese Medicine, Changchun, China

**Keywords:** hepatocellular carcinoma, RNA modification, m6A, m7G, m5C, m1A, m3C, ψ

## Abstract

Hepatocellular carcinoma (HCC) is a highly mortal type of primary liver cancer. Abnormal epigenetic modifications are present in HCC, and RNA modification is dynamic and reversible and is a key post-transcriptional regulator. With the in-depth study of post-transcriptional modifications, RNA modifications are aberrantly expressed in human cancers. Moreover, the regulators of RNA modifications can be used as potential targets for cancer therapy. In RNA modifications, N6-methyladenosine (m6A), N7-methylguanosine (m7G), and 5-methylcytosine (m5C) and their regulators have important regulatory roles in HCC progression and represent potential novel biomarkers for the confirmation of diagnosis and treatment of HCC. This review focuses on RNA modifications in HCC and the roles and mechanisms of m6A, m7G, m5C, N1-methyladenosine (m1A), N3-methylcytosine (m3C), and pseudouridine (ψ) on its development and maintenance. The potential therapeutic strategies of RNA modifications are elaborated for HCC.

## 1 Introduction

Hepatocellular carcinoma (HCC) is the fifth most prevalent cancer worldwide (Llovet et al., 2021). As an important histological subtype of liver cancer, HCC is characterized by high recurrence rates and heterogeneity and represents a major health challenge (Bosch et al., 1999). HCC has a poor prognosis, making it crucial to investigate the molecular mechanisms and therapeutic strategies underlying hepatocellular carcinogenesis (Llovet et al., 2008; Chen et al., 2015). It has been shown that hepatocellular carcinogenesis is regulated by complex genetic and epigenetic mechanisms with pathophysiological processes involving viral and non-viral factors and influenced by fatty liver, immune cell infiltration, and the tumor microenvironment (Ozen et al., 2013; Han et al., 2018; Pea et al., 2021; Braghini et al., 2022). A study using whole-genome and -exome sequencing analysis has shown that epigenetic regulation is the most unusual differential modifier in HCC, with aberrant RNA modifications leading to differential gene expression, affecting the HCC formation and progression (Schulze et al., 2015; Klingenberg et al., 2017; Chen et al., 2018). One of the characteristics of tumor cells is their dynamic adaptation to a microenvironment that is harmful to them. Therefore, modulating RNA modifications can dynamically regulate tumor cell proliferation and apoptosis, affecting the fate of both normal and cancerous cells (Haruehanroengra et al., 2020; Uddin et al., 2020).

Epigenetics is a branch in the field of genetics which explores the phenotypes that can be inherited from chromosomal changes which do not modify the nucleotide sequence of genes, of which RNA modification has been a recent hotspot of epigenetic research (Fu et al., 2014). In nature, dynamically created RNA modifications are widely present on all nucleotides (Teng et al., 2021; Willbanks et al., 2021). In the context of advances in high-throughput sequencing technologies, there has been a landmark breakthrough in the identification and characterization of human transcriptome modifications (Zhao et al., 2017). To date, over 170 different types of RNA modification have been detected, existing not only on transfer (tRNA), ribosomal (rRNA), and messenger (mRNA) RNA but also on other noncoding RNAs (ncRNAs) such as long-noncoding RNA (lncRNA) and microRNA (miRNA), and have direct or indirect functional effects on gene expression and regulate a wide range of biological activities (Boccaletto et al., 2018; Rong et al., 2021a). RNA modifications that have been subject to intensive study include N6-methyladenosine (m6A), N7-methylguanosine (m7G), 5-methylcytosine (m5C), N1-methyladenosine (m1A), N3-methylcytosine (m3C), and pseudouridine (ψ) (Delaunay and Frye, 2019; Zhang et al., 2021a). However, the biological functions and mechanisms of these RNA modifications continue to be discovered within the complex epigenomic transcriptome environment of HCC.

The epigenomic environment is highly complex. RNA modification is an important epigenetic regulatory mechanism, regulating the structure, function, and stability of RNA and controlling the expression of related genes (Roundtree et al., 2017). RNA modifications are highly relevant to biological factors including RNA decay, stability, metabolism, and binding to RNA-binding proteins (Shi et al., 2020a; Boo and Kim, 2020; Wang et al., 2021a). A large majority of works indicate that dysregulation of RNA epigenetic pathways is tightly linked to the pathogenesis of human diseases, particularly cancer (Delaunay and Frye, 2019; Yang et al., 2019; Barbieri and Kouzarides, 2020; Liang et al., 2020). Cancer is a type of disease featured by the progressive build-up of genetic and epigenetic modifications of unique cancer-causing and oncogenic genes that regulate cellular behaviors such as proliferation, differentiation, and migration. Indeed, dynamic RNA modifications are implicated in the metabolic RNA process, an important emerging regulator in cancer (Ozen et al., 2013; Delaunay and Frye, 2019; Han et al., 2021). Directing abnormal posttranscriptional modifications in carcinoma cells promises to be an effective tumor therapy (Zhao et al., 2014; Geula et al., 2015; Su et al., 2018; Janin et al., 2019; Uddin et al., 2020; Chen et al., 2021a; Han et al., 2021; Nombela et al., 2021). This review concentrates on the functions of six RNA modifications that regulate cancer progression in HCC.

## 2 N6-methyladenosine

**FIGURE 1:**
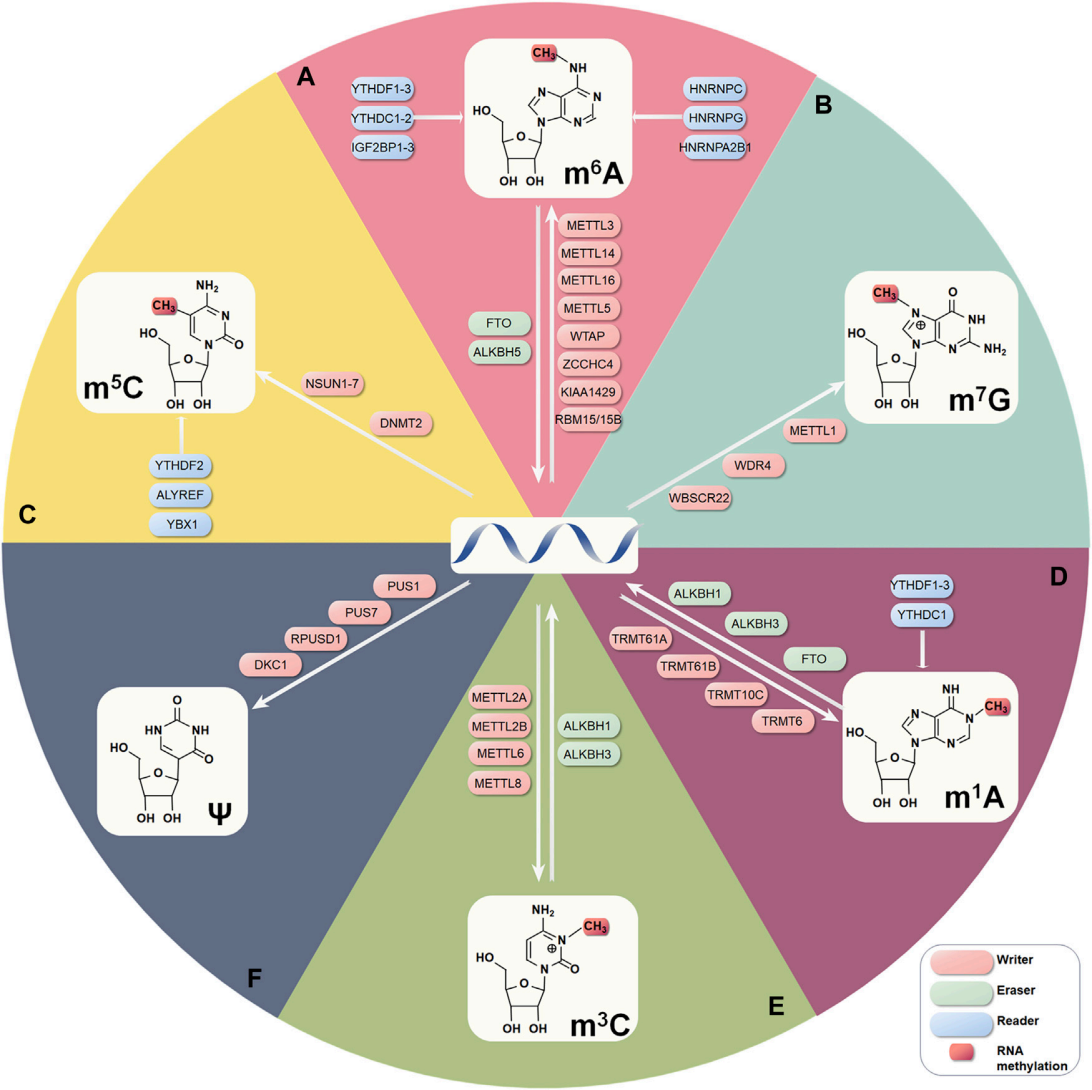
The six internal RNA modifications focused on in this review and their regulatory mechanisms. **(A)** m6A. **(B)** m7G. **(C)** m5C. **(D)** m1A. **(E)** m3C. **(F)** Ψ.

**FIGURE 2:**
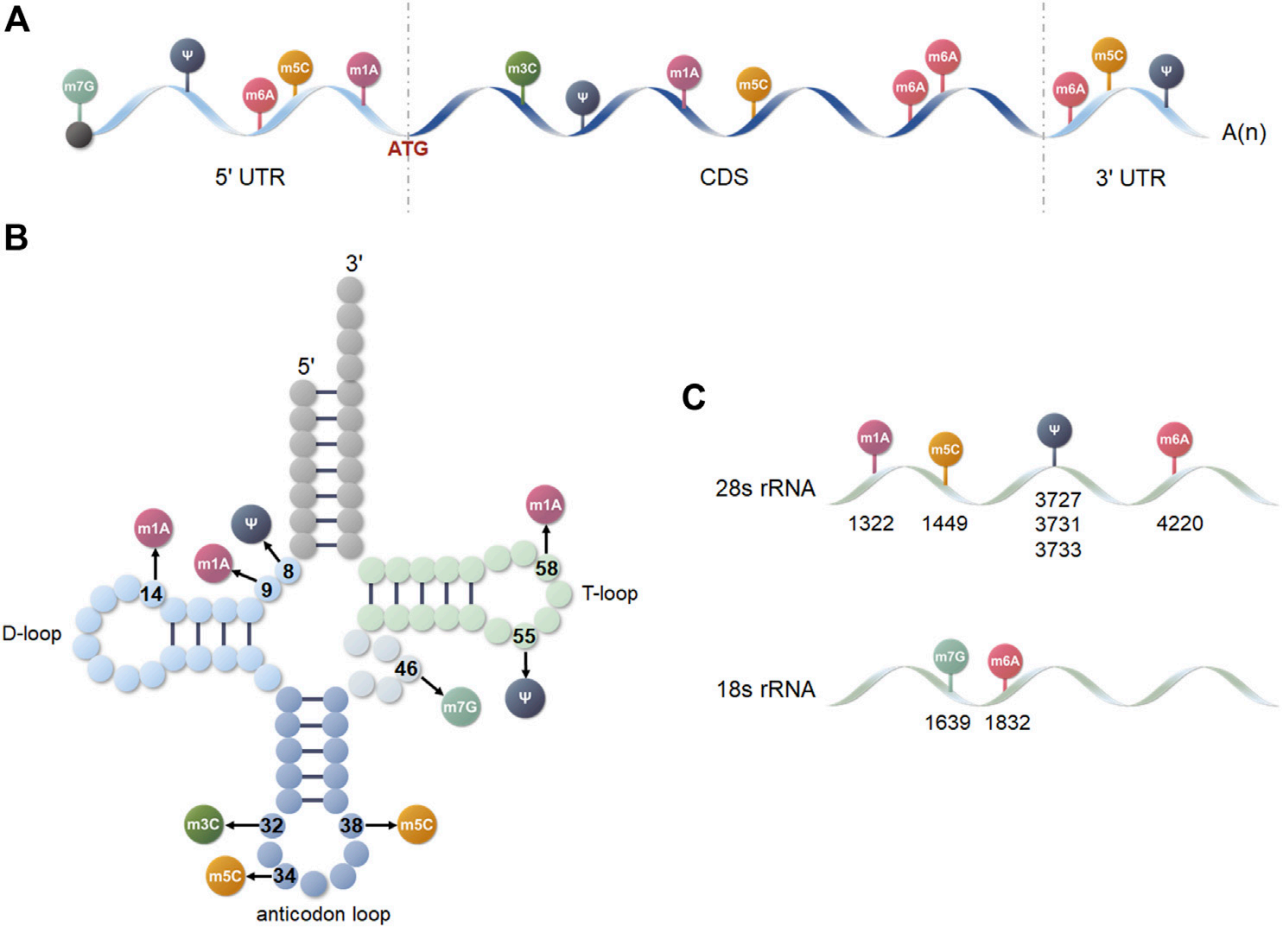
Distribution of the six posttranscriptional modifications on different RNA isoforms. **(A)** mRNA. **(B)** tRNA. **(C)** 18 and 28s rRNA.

First discovered in 1974, m6A is the most popular RNA modification (Figure 1A). m6A modifications are dynamically reversible and are primarily found in mRNA, rRNA, tRNA, miRNA, circular RNA (circRNA), lncRNA, and small nuclear RNA (snRNA), regulating molecular functions and participating in the pathogenesis of various diseases, including cancer (Figure 2) (Desrosiers et al., 1974; Krug et al., 1976; Horowitz et al., 1984; Björk et al., 1987; Shimba et al., 1995; Piekna-Przybylska et al., 2008; Wang and He, 2014; Alarcón et al., 2015; Ben-Haim et al., 2015; Kennedy et al., 2016; Patil et al., 2016; Bartosovic et al., 2017; Yang et al., 2017b; Coots et al., 2017; Rosa-Mercado et al., 2017; Zhou et al., 2017; Liu et al., 2018; Taketo et al., 2018; Yang et al., 2018; van Tran et al., 2019; Xiao et al., 2019; Zhang et al., 2020b; Di Timoteo et al., 2020). m6A modifications not only affect RNA stability, splicing, transcription, processing, translation, and metabolism, they regulate gene expression in a wide range of physiological processes and play an important role in cellular differentiation, apoptosis, and proliferation (Meyer et al., 2015; Yue et al., 2015; Yoon et al., 2017; Zhao et al., 2017; Chen et al., 2018).

### 2.1 N6-methyladenosine high-throughput sequencing methods

Accurate detection of RNA modifications is the basis of epigenomic studies, and several types of assays for m6A modifications have been developed. Antibody-based assays include methylated RNA immunoprecipitation sequencing (m6A-MeRIP-seq) and m6A-level and isoform-characterization sequencing (m6A-LAIC-seq), enabling measurement of transcriptome-wide m6A modification levels (Dominissini et al., 2012; Meyer et al., 2012; Linder et al., 2015; Molinie et al., 2016). The digestion-based sequencing assays MazF RNase assisted sequencing (MAZTER-seq) and m^6^A-sensitive RNA-endoribonuclease–facilitated sequencing (m6A-REF-seq) are simple and efficient (Zhang et al., 2019b; Garcia-Campos et al., 2019; Pandey and Pillai, 2019). Ligation-based detection methods are highly sensitive and low cost, including single-base elongation- and ligation-based qPCR amplification method (SELECT) and locus-specific extension of annealed DNA probes targeting m6A and sequencing (LEAD-m6A-seq) (Xiao et al., 2018; Wang et al., 2021d). Deaminase sequencing adjacent to RNA modification targets (DART-seq) is an antibody-free m6A sequencing method based on gene editing (Meyer, 2019). In addition, the DART-seq-based single-cell assay (scDART-seq) is the first method to examine m6A sites in single cells. Nanopore RNA sequencing (RNA-seq) is a real-time sequencing technique that is simple and sensitive, greatly expanding the scope of m6A research and providing a scientific platform for the quantitative detection of m6A modifications (Garalde et al., 2018). However, due to the still high error rate of nanopore sequencing itself and the relatively small number of RNA methylation modifications, which may be below the threshold level, the detection is prone to have a high false positive rate (Leger et al., 2021). The detection of RNA modifications at single molecule level with single nucleotide resolution can be chosen as a classical sequencing tool to ensure the accuracy of detection. Overall, these m6A sequencing methods could provide potential clues for HCC diagnosis and treatment.

### 2.2 N6-methyladenosine enzymes

The m6A methylation modification is written by methyltransferase 3 (METTL3), 14 (METTL14), and 16 (METTL16), RNA binding motif proteins 15 (RBM15) and 15B (RBM15B), KIAA1429, and Wilms tumor 1-associated protein (WTAP). It is subsequently erased by the demethylases alkane hydroxylase homolog 5 (ALKBH5) and fat mass and obesity-associated protein (FTO). Proteins that read the modification include members of the YT521-B homolog (YTH) structural domain family of m6A RNA binding proteins (YTHDF1, YTHDF2, YTHDF3, YTHDC1, and YTHDC2), insulin-like growth factor 2 mRNA-binding protein family (IGF2BP1, IGF2BP2, and IGF2BP3), and heterogeneous nuclear ribonucleoprotein family (HNRNPA2B1, HNRNPC, and HNRNPG), and proline-rich and coiled-coil protein 2A (PRRC2A) (Schwartz et al., 2014b; Fu et al., 2014; Meyer and Jaffrey, 2014; Haussmann et al., 2016; Schumann et al., 2016; Schwartz, 2016; Arguello et al., 2017; Edupuganti et al., 2017; Meyer and Jaffrey, 2017; Wu et al., 2017; Müller et al., 2019). m6A methyltransferases and demethylases are associated with various diseases, including cancer (Boissel et al., 2009; Church et al., 2010; Boles and Temple, 2017; Wang et al., 2018; Chen et al., 2019b).

The addition of m6A onto mRNA, long intergenic non-coding RNA (lincRNA), and miRNA is catalyzed by the METTL3-METTL14 complex (Liu et al., 2014; Wang et al., 2016b). METTL3 is the central element of catalysis that converts adenosine to m6A through its methyltransferase structural domain, enhances mRNA translation, and affects gene expression and the pattern of alternative splicing (Dominissini et al., 2012; Schumann et al., 2016; Bartosovic et al., 2017; Ma et al., 2017; Rosa-Mercado et al., 2017). METTL14 recognizes RNA substrates as well as recruits microprocessor complexes for co-transcription on primary (pri)-miRNA transcripts (Huang et al., 2019). The combination of the two methyltransferases improves the methylation rate and has a cooperative influence, preferentially methylating RNA primers to contain the consensual sequence GGACU (Bokar et al., 1994; Liu et al., 2014; Wang et al., 2014; Wang et al., 2016a; Wang et al., 2016b; Ke et al., 2017). WTAP is the third key member as a regulatory subunit of the mammalian m6A methyltransferase enzyme complex, enhancing methyltransferase catalytic activity (Horiuchi et al., 2006; Ping et al., 2014). Indeed, the major m6A mediators have a significant role in cancer causation or inhibition in different cancer types. METTL3 is shown to be highly explored in advanced acute myeloid leukemia (AML) and has reported roles in lung cancer and HCC (Lin et al., 2016; Vu et al., 2017; Chen et al., 2018). METTL16 was shown to be required for HCC cell proliferation, while METTL3 and METTL14 have conflicting roles in HCC (Vu et al., 2017; Chen et al., 2018).

The addition of m6A is reversible with reliance on a coordinated and highly dynamic system of methyltransferases and demethylases. m6A demethylases FTO and ALKBH5 are part of the non-heme Fe(II)/α-ketoglutarate-dependent dioxygenase family (Gerken et al., 2007; Fu et al., 2010; Jia et al., 2011; Zheng et al., 2013). FTO is related to human obesity and mental development and was the first m6A mRNA demethylase identified to convert mRNA m6A to adenosine in mRNA (Dina et al., 2007; Loos and Yeo, 2014; Mauer et al., 2017; Wei et al., 2018). ALKBH5 reverses m6A to adenosine in a direct manner and influences mRNA export and RNA turnover in a demethylation-dependent way (Zheng et al., 2013). Indeed, FTO is a key factor regulating HCC, promoting migration, invasion, and proliferation of HCC cells, countering the function of ALKBH5 in HCC(Fischer et al., 2009; Ueda et al., 2017).

m6A has a major role in cell differentiation with tissue development. It can regulate the entire RNA lifecycle, in terms of RNA processing, decay, and translation, through the identification of specific binding proteins, dynamically regulating physiological and pathological processes such as tumorigenesis (Allis and Jenuwein, 2016; Shi et al., 2017; Chen et al., 2019b; Han et al., 2019b; Wu et al., 2019b). m6A writers have a common RNA-binding YTH structural domain, including YTHDC1, YTHDC2, YTHDF1, YTHDF1 YTHDF2, and YTHDF3 (Li et al., 2014; Xu et al., 2014). Eukaryotic initiation factor 3 (eIF3), HNRNPC, HNRNPA2B1, IGF2BP1, IGF2BP2, IGF2BP3, PRRC2A, and fragile X mental retardation protein (FMRP) also proved to be m6A writers (Liu et al., 2015; Liu et al., 2017). YTH structural domain proteins recognize m6A modifications specifically and manage mRNA decay, translation, and maturation (Liu et al., 2017). Heterodimers of YTH structural domain family (YTHDF) proteins are mainly found in the cytoplasm, while m6A affects mRNA decay upon the combination of three YTHDF members (Zaccara and Jaffrey, 2020).

m6A promotes the binding of regulatory proteins by altering the structure of RNA to regulate gene expression and RNA maturation (Wan et al., 2014). The IGF2BPs family of m6A readers specifically recognize the GG (m6A) C motif through its K homology (KH) structural domain, promote the structured stability and translations in most target mRNAs, and are highly expressed in various cancers and involved in many different molecular mechanisms (Huang et al., 2018). IGF2BPs act as m6A readers during post-transcriptional gene regulation and the biology of cancer (Huang et al., 2018).

There is growing evidence suggesting that abnormal changes in the levels of total m6A and its regulators in HCC tissues are strongly connected with poor clinical survival (Chen et al., 2020; Li et al., 2021c). m6A regulators of the modifications perform oncogenic or anti-tumor effects in HCC by influencing the expression of particular genes.

### 2.3 Role of N6-methyladenosine in hepatocellular carcinoma

**FIGURE 3:**
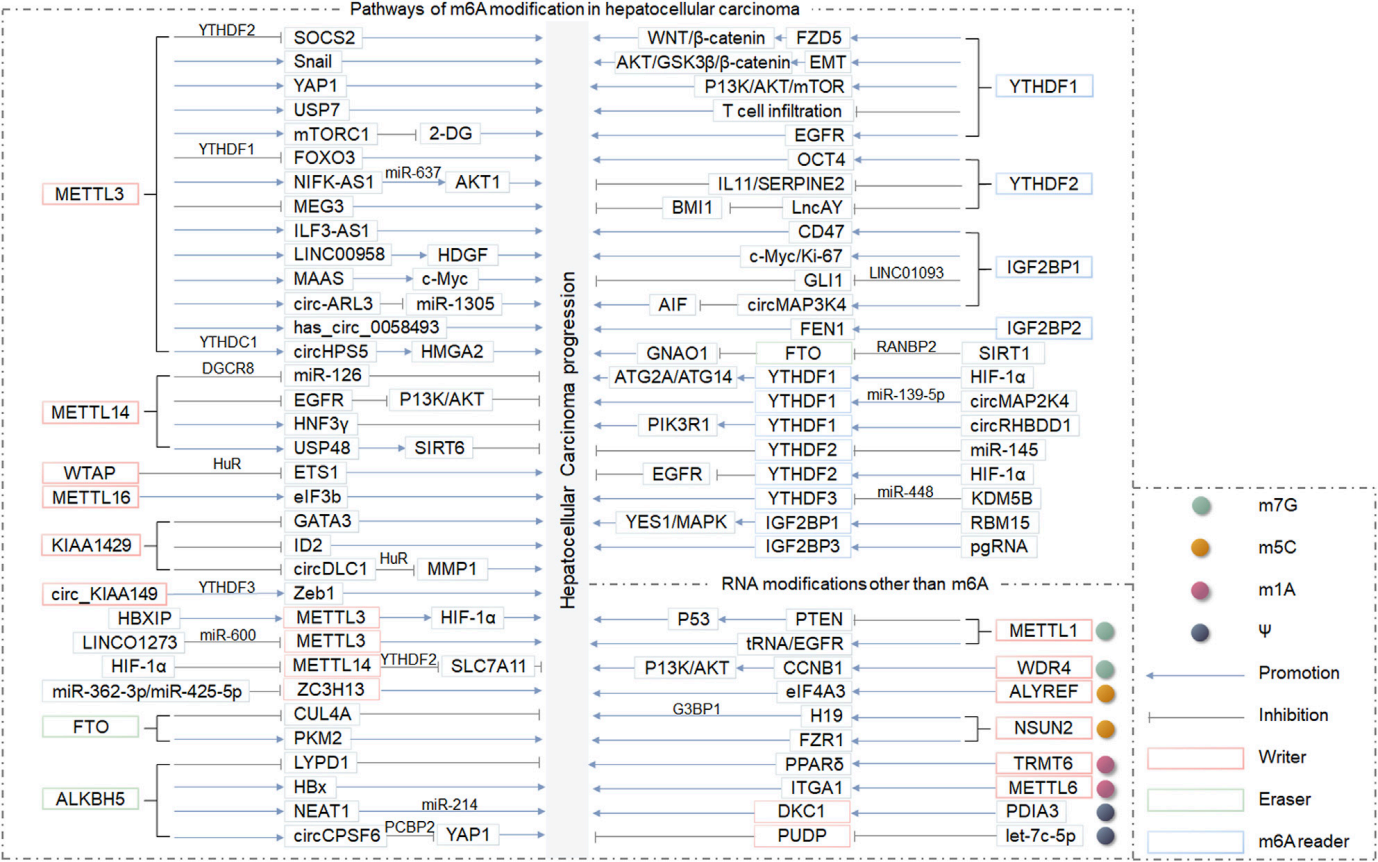
The role of RNA epigenetic modifications in HCC progression.

m6A and its regulators are closely related to oncogenic or tumor suppressor functions and play a role as oncogenes or antioncogenes in malignant tumors (Zhou et al., 2015; Lin et al., 2016; Li et al., 2017b; Xiang et al., 2017; Zhong et al., 2018; Han et al., 2019a). With the intensive study of HCC, m6A modifiers and regulators have been found to take a pivotal role in the development and progression of HCC, capable of regulating HCC cell phenotype, migration, invasion, and epithelial-mesenchymal transition (EMT) processes, and a potential therapeutic target for HCC treatment. (Figure 3). Many studies have provided data to support the expression of m6A modifications and m6A regulators in HCC *via* bioinformatics analysis (Liu et al., 2020a; Jiang et al., 2021). METTL3, YTHDF1, YTHDF2, YTHDF3, YTHDC1, YTHDC2, FTO, KIAA1429, HNRNPC, HNRNPA2B1, and RBM15 are all overexpressed in HCC (Liu et al., 2020a). One study found higher expression levels of FTO, ALKBH5, RBM15, and WTAP in HCC through the TCGA database (Jiang et al., 2021). Copy number variation (CNV), mutation, and clinical databases of HCC patients from The Cancer Genome Atlas (TCGA) showed differential expression of the m6A regulators in HCC, significantly associated with clinicopathological features, and correlated with sorafenib treatment effect (Jiang et al., 2021). Patients with m6A regulator mutations showed inferior overall survival (OS) and disease-free survival (DFS).

#### 2.3.1 Writers

METTL3 is highly expressed in HCC, METTL3 regulates RNA expression in HCC in an m6A-dependent manner and is independent of recurrence-free survival (RFS) as a prognostic factor, promoting HCC cell proliferation, colony formation, migration, and tumorigenicity *in vitro* (Supplementary Table S1) (Liu et al., 2020d; Qu et al., 2020). METTL3 could serve as a potential HCC prognostic biological marker and contribute to HCC carcinogenesis, tumor progression, and drug resistance (Chen et al., 2018; Liu et al., 2021a; Pan et al., 2021). Studies have shown that overexpression of promotes HCC progression by increasing the level of m6A modification and suppressing the expression of cytokine signaling 2 (SOCS2) in HCC by a YTHDF2-dependent mechanism (Chen et al., 2018).

HCC correlates with ubiquitination of METTL3, METTL3 SUMOylation was increased in HCC, and the UBC9/SUMOylated METTL3/Snail axis is a novel mediator of sumoylation (SUMO) pathway involvement in HCC progression (Xu et al., 2020). In addition, inhibition of METTL3 to inhibit glycolysis is also a potential strategy for HCC treatment (Lin et al., 2020a). Another study showed that overexpression of METTL3 promoted invasion, migration, and proliferation of HCC cells by upregulating USP7 expression through m6A methylation (Li et al., 2021d). Furthermore, hepatitis B virus X-interacting protein (HBXIP)-mediated METTL3 upregulation driven by hypoxia-inducible factor 1-α (HIF-1α) promotes metabolic reprogramming in HCC cells (Yang et al., 2021).

Sorafenib is a first-line drug approved for treating advanced HCC. However, sorafenib resistance reduces its efficacy in HCC(Vogel et al., 2021). METTL3 is found to be significantly decreased in sorafenib-resistant HCC, and depletion of METTL3 under conditions of hypoxia promotes sorafenib tolerance and angiogenic gene expression in HCC cells and activates autophagy-related pathways (Lin et al., 2020b). Overexpression of forkhead box O3 (FOXO3) rescues the METTL3 depletion-induced sorafenib resistance phenotype. FOXO3 is an essential marker of m6A modification in HCC in its resistance to sorafenib (Lin et al., 2020b). lincRNA 1,273 (LINC01273) is significantly overexpressed in sorafenib-resistant HCC tissues, binds complementarily to miR-600, promotes miR-600-targeted downregulation of METTL3 expression levels, reduces m6A modification in HCC, and promotes HCC progression. The LINC01273/miR-600/METTL3 feedback regulatory axis is a promising intervention pathway for patients with sorafenib-resistant HCC (Kong et al., 2022).

The METTL3/IGF2BP1 complex enhanced the stability of NIFK-AS1 mRNA by increasing the expression level of m6A, promoted the growth and invasion of HCC cells, and promoted the progression of HCC through the AKT1-MMP7/9 axis (Chen et al., 2021d). In addition, METTL3-mediated m6A modification down-regulates the expression level of maternally expressed 3 (MEG3), which targets miRNA 544b (miR-544b), miR-544b promotes HCC proliferation, invasion, and metastasis by suppressing BTG2 expression (Wu et al., 2021b). Interleukin enhancer-binding factor 3 (IlF3) antisense RNA 1 (ILF3-AS1) promotes HCC cell proliferation, migration, and invasion increases ILF3 m6A levels in an m6A-dependent manner *via* METTL3, and is an important oncogene in HCC progression, providing a new therapeutic target for HCC (Bo et al., 2021). METTL3 mediates m6A modification to upregulate the expression of lincRNA 958 (LINC00958), which promotes HCC production and development by binding to miRNA 3619-5p (miR-3619-5p) to upregulate the expression of HCC growth factor (HDGF) (Zuo et al., 2020). Moreover, METTL3 accelerates HCC progression by upregulating the m6A level of circRNA hsa_circ_0058493 and promoting its binding to YTHDC1 (Wu et al., 2021a). METTL3 mediates the expression of m6A to promote the formation of circRNA HPS5 (circHPS5), which can act as a miRNA 370 (miR-370) sponge to regulate the expression of high-mobility group AT-hook 2 (HMGA2) and further accelerate tumorigenesis in HCC cells, providing a potential prognostic marker and therapeutic target (Rong et al., 2021b). Hepatitis B virus (HBV) infection is one of the main risk elements for HCC. METTL3 mediates lncRNA mitogen-activated protein kinase activation of protein kinase five antisense RNA 1 (MAAS) targeting c-Myc to promote the proliferation of HBV + HCC cells (Tao et al., 2022). It has also been shown that HBV x protein (HBx) upregulates METTL3 expression, increases m6A modification of circRNA ARL3 (circ-ARL3), and circ-ARL3 antagonizes the repressive effect of miRNA 1,305 (miR-1305) on oncogenes, thereby promoting HBV + HCC progression, and targeting this pathway is a promising approach for treating HBV + HCC patients (Rao et al., 2021).

*METTL14* is a major gene for aberrant m6A modification that is downregulated and can act as a poor element for recurrence-free survival associated with tumor metastasis in HCC (Ma et al., 2017; Shi et al., 2020c). Overexpression of METTL14 inhibits HCC malignant progression by upregulating m6A expression levels of genes critical for prognosis in HCC patients, such as cysteine sulfite decarboxylase, glutamate-oxaloacetate transaminase 2, and cytokine signaling inhibitor 2 (Li et al., 2020). METTL14 is lowly expressed in HCC, and overexpression of METTL14 is able to promote m6A by increasing the binding of the microprocessor protein DiGeorge syndrome critical region 8 (DGCR8) and pri-miR126 to inhibit HCC progression (Ma et al., 2017). METTL14 regulates the epidermal growth factor receptor (EGFR)/phosphoinositide 3-kinase (PI3K)/AKT signaling pathway to inhibit migration, invasion, and EMT of HCC cells (Shi et al., 2020c). METTL14 mediates downregulation of hepatocyte nuclear factor 3γ (HNF3γ) expression levels, promotes cell differentiation to inhibit HCC progression, and is sensitive to sorafenib-induced growth inhibition and apoptosis in HCC cells (Zhou et al., 2020). Hypoxia triggers METTL14 inhibition in a HIF-1α-dependent manner, effectively eliminating iron death in HCC cells. METTL14 induces degradation of solute carrier family seven member 11 (SLC7A11) in a YTHDF2-dependent pathway, and the HIF-1α/METTL14/YTHDF2/SLC7A11 axis is a potential therapeutic target for HCC interventional embolization therapy. therapeutic target (Fan et al., 2021b). Additionally, METTL14-mediated m6A modification maintains sirtuin 6 (SIRT6) stability by regulating the expression of ubiquitin-specific peptidase 48 (USP48) and promoting the tumor suppressive function of glycolysis to regulate the metabolic activity of HCC (Du et al., 2021).

WTAP is expressed at a high level in HCC, promotes HCC development, is associated with poor prognosis, and is shown to be an independent predictor for survival in HCC (Chen et al., 2019c). Knockdown of WTAP can upregulate and promote autophagy in HCC, promotes liver kinase B1 (LKB1) expression, increases adenosine 5′monophosphate-activated protein kinase (AMPK) phosphorylation, and inhibits HCC proliferation (Li et al., 2021a). WTAP also promotes the proliferative capacity and growth of HCC cells (Chen et al., 2019c). ETS proto-oncogene 1 (ETS1) is a downstream effector of WTAP and WTAP-regulated m6A modification leads to post-transcriptional repression of ETS1, which promotes HCC progression through the human antigen R (HuR)-ETS1-p21/p27 axis and is a potential target for HCC therapy (Chen et al., 2019c). Zinc finger CCCH domain-containing protein 13 (ZC3H13) is a potential HCC target for biomarker and therapy, is significantly downregulated in HCC, and downregulation of ZC3H13 mediated by miRNA 362-3p (miR-362-3p) and miRNA 5425-5p (miR-425-5p) is associated with poor prognosis and cancer immune invasion in HCC (Wu et al., 2022). As the largest known element of the m6A methyltransferase, KIAA1429 is shown to be highly upregulated in HCC tissues and related to poor prognosis in HCC patients (Cheng et al., 2019; Lan et al., 2019). KIAA1429 promotes the malignant phenotype of HCC cells by inducing m6A methylation of GATA binding protein 3 (GATA3) to downregulate its expression, thus providing a new strategy for HCC treatment (Lan et al., 2019). In addition, KIAA1429 inhibits the inhibitor of DNA binding 2 (ID2) mRNA by upregulating its m6A modification, promoting HCC migration and invasion (Cheng et al., 2019). Overexpression of circ_KIAA1429 promotes HCC migration, invasion, and EMT processes and accelerates HCC progression through the m6A/YTHDF3/Zinc finger E box binding homeobox1 (ZEB1) pathway (Wang et al., 2020a). It has been shown that circRNA DLC1 (circDLC1) is downregulated in HCC tissues, and knockdown of KIAA1429 is able to increase the expression level of circDLC1 and reduce the interaction between HuR and matrix metallopeptidase 1 (MMP1), thereby inhibiting MMP1 expression and ultimately contributing to the inhibition of HCC progression (Liu et al., 2021b). Recent research has shown that is aberrantly overexpressed in HCC, is associated with poor prognosis, promotes tumorigenesis by regulating eIF3a/b expression, and could be a potential new target for HCC therapy (Su et al., 2022).

#### 2.3.2 Erasers

Reduced expression of FTO in HCC cells and tissues leads to the accumulation of m6A in HCC and is associated with poor patient prognosis, and it may serve as a novel biomarker for HCC (Zhao et al., 2019a). One study demonstrated that overexpression of FTO was able to block the proliferation of HCC cells and inhibit HCC progression by targeting the downregulation of CUL4A expression (Mittenbühler et al., 2020). Deacetylase silencing information regulator 1 (SIRT1) promotes HCC progression by downregulating the expression of FTO, increasing the expression level of m6A, and decreasing the expression level of G protein subunit αO1 (*GNA O 1*) (Liu et al., 2020b). Transarterial chemoembolization (TACE) has dramatically improved the OS of some patients with unresectable HCC (Liu et al., 2021c). A study found that YTHDC2 expression levels were upregulated in HCC tissues while FTO expression levels were decreased, and functional single nucleotide polymorphisms (SNPs) in *YTHDC2* and *FTO* were found to have prognostic value in TACE-treated HCC patients (Liu et al., 2021c).

*ALKBH5* expression is downregulated in HCC and inhibits the proliferation as well as the invasive ability in HCC cells, which is an indicator of an independent prognosis in HCC patients. ALKBH5-mediated m6A demethylation in HCC cells attenuates LY6/PLAUR domain containing 1 (LYPD1) expression and inhibits the malignant progression of HCC (Chen et al., 2020). In HBV-HCC tissues, depletion of ALKBH5 significantly inhibited HBV-driven tumor cell growth and migration. HBV could upregulate ALKBH5 through the HBx-WDR5-H3K4me3 axis and promote the progression of HCC (Fang and Chen, 2020; Qu et al., 2021). ALKBH5 can upregulate nuclear paraspeckle assembly transcript 1 (NEAT1) expression by inhibiting m6A enrichment, and NEAT1 promotes cell proliferation and migration of HCC by sponging miRNA 214 (miR-214) (Yeermaike et al., 2022). ALKBH5 mediates circular cleavage and polyadenylation-specific factor 6 (circCPSF6) demethylation, promoting the malignant development of HCC through aberrant activation of the circCPSF6 and Yes1 associated transcriptional regulator (YAP1) axis, providing new insights into the regulation of circRNAs by m6A modification together with epigenetic reprogramming in HCC (Chen et al., 2022). Both FTO and ALKBH5 may serve as potential targets for the treatment of HCC. Although the above studies demonstrated that ALKBH5 can be a therapeutic target for HCC and overexpression of ALKBH5 can inhibit the progression of HCC. However, other studies have shown that ALKBH5 is involved in DNA repair and that overexpression of ALKBH5 can promote DNA alkylation repair (Akula et al., 2021). Since DNA repair may prolong the telomere length of cancer cells and allow them to continue to proliferate, there is a need to balance the therapeutic effects of ALKBH5 on cancers such as HCC with the promotion of cancer cells through DNA repair (Roake and Artandi, 2016). Anticancer agents that target the DNA repair pathway, such as PARP inhibitors, have been developed to promote apoptosis in cancer cells by inhibiting DNA damage repair in cancer cells (Gavande et al., 2016; Huang and Zhou, 2021). This type of inhibitor can be utilized synergistically with ALKBH5 to enhance the efficacy of targeting ALKBH5 for the treatment of HCC.

#### 2.3.3 Readers

The expression of *YTHDF1* was elevated in HCC and showed a positive correlation with the pathological HCC stage (Zhao et al., 2018a). Knockdown YTHDF1 remarkably inhibited the proliferation, migration, and invasion of HCC (Zhao et al., 2018a).

YTHDF1 mediates frizzled class receptor 5 (*FZD5*) expression to regulate the WNT/β-catenin axis to exert oncogenic function as a potential HCC therapeutic strategy (Liu et al., 2020e). YTHDF1 promotes the malignant phenotype of HCC by activating AKT/glycogen synthase kinase (GSK)/3β/β-catenin signaling and promoting EMT (Bian et al., 2020). YTHDF1 can also promote HCC progression by activating the PI3K/AKT/mammalian target of rapamycin (mTOR) signaling route to induce EMT (Luo et al., 2021). It has been shown that overexpression of YTHDF1 depends on high expression of m6A modifications in HCC, inhibits cellular infiltration of CD3^+^ and CD8^+^ T, and promotes malignant progression of HCC (Li et al., 2021b). YTHDF1 deficiency inhibits HCC metastasis, growth, and autophagy (Li et al., 2021c). HIF-1α-regulated *YTHDF1* expression enhanced the translation of autophagy-associated 2A (ATG2A) and 14 (ATG14) in an m6A-dependent manner to drive hypoxia-induced HCC autophagy It was shown that radiofrequency ablation (IRFA)-deficient sublethal heat stress promotes HCC progression by upregulating EGFR expression levels through an N6-methyladenosine mRNA methylation-dependent mechanism (Su et al., 2021). The m6A/YTHDF1/EGFR axis promotes HCC progression after IRFA, providing a rationale for YTHDF1 binding to EGFR to regulate HCC progression (Su et al., 2021). Furthermore, circRNA MAP2K4 (circMAP2K4) was validated to promote HCC cell proliferation by binding with miRNA 139-5p (miR-139-5p) to promote YTHDF1 expression, providing clues for HCC therapeutic targets (Chi et al., 2021). Moreover, circRNA RHBDD1 (circRHBDD1) uses YTHDF1 to accelerate the translation of phosphoinositide-3-kinase regulatory subunit 1 (PIK3R1) in an m6A-dependent manner and plays an important role in the metabolism of HCC. Inhibition of circRHBDD1 enhances the treatment of HCC with anti-programmed cell death protein 1 (PD-1) (Cai et al., 2022). Thus, YTHDF1 is a potential biomarker of prognosis and a target for treatment (Li et al., 2021c).

YTHDF2 is negatively correlated with survival in HCC patients and YTHDF2 upregulates octamer-binding transcription factor 4 (OCT4) expression in an m6A-dependent manner, promoting HCC progression (Zhang et al., 2020a). miRNA 145 (miR-145) regulates m6A expression levels by regulating the 3′-UTR of YTHDF2 mRNA in HCC cells to regulate m6A expression levels (Yang et al., 2017c). It was shown that hypoxia-specific downregulates *YTHDF2* expression in HCC cells and YTHDF2 binding to the m6A site modified by EGFR 3′-UTR promotes EGFR mRNA degradation in HCC cells and inhibits proliferation of HCC cells (Zhong et al., 2019). YTHDF2 can regulate serpin family E member 2 (SERPINE2), interleukin 11 (IL11)decay, and vascular abnormalities in HCC, and inhibits the malignant progression of HCC (Hou et al., 2019). It was shown that YTHDF2 inhibited the expression of lncRNA AY (lncAY) and suppressed HCC progression through the BMI1 proto-oncogene polysaccharide ring finger (BMI1)/WNT/β-catenin axis form (Chen et al., 2021b). It was also shown that lysine demethylase 5B (KDM5B) regulates the axis formed by YTHDF3 and integrin subunit alpha 6 (ITGA6) axis by suppressing miRNA 448 (*miR-448*) expression and promoting HCC progression (Guo et al., 2021).

RBM15 is highly expressed in HCC, and RBM15 promotes HCC progression through the IGF2BP1/YES1/MAPK axis (Cai et al., 2021). Microwave ablation is a therapeutic approach for HCC, and studies have shown that sublethal heat treatment promotes HCC migration and EMT transformation through induction of METTL3 and CD47, that METTL3 mediates HCC migration through CD47 in an m 6 A-dependent manner, and that the METTL3/IGF2BP1/CD47 axis is a target for potential treatment by microwave ablation, providing clues to the link between RNA methylation and incomplete thermal ablation (Fan et al., 2021a). It was shown that IGF2BP1 can enhance c-Myc and Ki-67 protein translation and promote HCC progression by binding and stabilizing proliferation markers C-Myc and Ki-67 (MKI67) (Gutschner et al., 2014). LincRNA 1,093 (LINC01093) inhibits HCC progression by promoting attenuation of GLI family zinc finger 1 (*GLI1*) mRNA through interaction with IGF2BP1 (He et al., 2019). The oncogene *IGF2BP2* is highly expressed in HCC and can be used for prognosis prediction (Pu et al., 2020). IGF2BP1 promotes the translation of circRNA MAP3K4 (circMAP3K4) into circMAP3K4-455aa, inhibiting allograft inflammatory factor 1 (AIF1) cleavage and nuclear distribution, preventing cisplatin-induced apoptosis, and promoting HCC progression (Duan et al., 2022). It has also been shown that pre-genomic RNA (pgRNA) promotes cancer progression in HCC and upregulates *IGF2BP3* expression at the post-transcriptional level (Ding et al., 2021). The above studies suggest that m6A-modification writers, erasers, and readers are involved in HCC progression and that regulator-mediated m6A modifications can influence HCC progression.

## 3 N7-methylguanosine

m7G modifications are commonly found in eubacteria, eukaryotes, and some archaea (Zhang et al., 2019a; Wiener and Schwartz, 2021) and are one of the few methylation modifications that introduce positively charged or amphiphilic ions into nucleobases (Figure 1B). m7G has a broad impact on mRNA, tRNA, and rRNA, playing a crucial function in various biological processes including transcriptional elongation, pre-mRNA splicing, and mRNA translation, as well as serving as an important indicator for disease diagnosis (Figure 2) (Alexandrov et al., 2002; Leulliot et al., 2008; Haag et al., 2015a; Lin et al., 2018; Liu et al., 2020c; Song et al., 2020; Blersch et al., 2021; Zhao et al., 2021).

### 3.1 N7-methylguanosine high-throughput sequencing methods

Precise identification of RNA modification sites is essential to investigate their functional effects in the control of gene expression and to clarify their relevance in different physiological processes (Cao et al., 2021). In response to the easy conversion of m7G to abasic sites, single-base sequencing methods have been developed to detect m7G in RNA (Boulias and Greer, 2019). Mainly includes tRNA reduction and cleavage sequencing (TRAC-seq)、AlkAline-seq、m7G-MAP-seq、m7G-seq and m7G individual-nucleotide-resolution cross-linking and immunoprecipitation with sequencing (m7G-miCLIP-seq) (Lin et al., 2018; Zhang et al., 2019a; Lin et al., 2019; Malbec et al., 2019; Marchand et al., 2021). These sequencing methods provide a scientific platform for detecting the expression levels of m7G modifications.

### 3.2 N7-methylguanosine enzymes

The main enzymes that perform m7G modification on RNA are methyltransferase-like 1 (METTL1), WD repeats structural domain 4 (WDR4), and Williams-Beuren syndrome chromosome region 22 protein (WBSCR22, also known as BUD23) (Deng et al., 2020). m7G modification of mRNA, tRNA, and miRNA is primarily mediated by METTL1 and WDR4, while WBSCR22 mediates m7G modification of rRNA (Létoquart et al., 2014). METTL1 adds m7G modifications in GA-rich sequences in mammalian internal mRNAs (Chen et al., 2021e; Zhao et al., 2021). In tRNAs, m7G is added by METTL1 and WDR4 in the AG (m7G)H motif (Malbec et al., 2019). There is growing evidence that impaired tRNA m7G modification is associated with various diseases (Braun et al., 2018). m7G tRNA modification mediated by METTL1 and WDR4 has critical functions in determining cell fate and growth (Lin et al., 2018). (Michaud et al., 2000; Shaheen et al., 2015; Lin et al., 2018; Ma et al., 2021; Orellana et al., 2021). A recent study showed that dysregulation of METTL1 is associated with viability and sensitivity to chemotherapy in colon and cervical cancer cells, suggesting a potential link between m7G tRNA modifications and cancer development (Cartlidge et al., 2005; Okamoto et al., 2014; Liu et al., 2019). METTL1 regulates cell proliferation, with potentially oncogenic effects on tRNA but tumor-suppressive activity in miRNA maturation, and is amplified explicitly in HCC and is correlated with poor prognosis (Pandolfini et al., 2019).

### 3.3 Role of N7-methylguanosine in hepatocellular carcinoma

Studies of m7G have shown that m7G modifications and its regulators METTL1, WDR4, and WBSCR22 are significantly upregulated in HCC and are associated with poor prognosis and chemotherapy resistance (Figure 3). Studies have shown that WBSCR22 is highly expressed in HCC and promotes the proliferation of HCC cells, making it a possible therapeutic target for HCC (Stefanska et al., 2014). Another study showed that *METTL1* is shown to be upregulated in HCC and promotes HCC migration and proliferation through the phosphatase and tensin homolog (PTEN) as well as the AKT signaling pathway (Tian et al., 2019). METTL1 exerts oncogenic activity by inhibiting PTEN signaling, and the METTL1/PTEN axis has potential for the treatment of HCC. METTL1-mediated tRNA m7G modification has also been proved to accelerate the translation of target mRNAs with higher m7G-associated codon frequencies, accelerating HCC progression and tumorigenesis (Chen et al., 2021e). Another study showed that upregulated *WDR4* expression significantly increased m7G methylation levels in HCC and conferred a poor prognosis. WDR4 can enhance HCC progression by promoting cyclin B1 (CCNB1) mRNA stability and translation and is a candidate HCC therapeutic target (Xia et al., 2021). These findings suggest that RNA m7G methylation modifications mediated by METTL1, WDR4, and WBSCR22 have important roles in HCC.

## 4 5-methylcytosine

The RNA 5-methylcytosine (m5C) is mainly distributed in archaea, prokaryotes, and eukaryotes, but is only conserved in *Saccharomyces cerevisiae* (Figure 1C) (Brzezicha et al., 2006; Edelheit et al., 2013; Chen et al., 2021c). It occurs in mRNA, tRNA, rRNA, ncRNA, and enhancer RNA (eRNA), regulating RNA in metabolic procedures, in terms of RNA translation, structure, stability, and mRNA export (Figure 2) (Haag et al., 2015b; Bourgeois et al., 2015; Tuorto et al., 2015; Haag et al., 2016; Amort et al., 2017; Yang et al., 2017a; Natchiar et al., 2017; García-Vílchez et al., 2019; Van Haute et al., 2019).

### 4.1 5-methylcytosine high-throughput sequencing methods

In recent years, advances in m5C detection methods have led to more extensive analysis of m5C. m5C-RIP-seq is an RNA immunoprecipitation method based on m5C antibodies (Edelheit et al., 2013). miCLIP-seq is a single nucleotide resolution protein-based method of immunoprecipitation (Hussain et al., 2013). AZA-immunoprecipitation-based sequencing (AZA-IP-seq) is a single-nucleotide method based on RCMT antibodies and 5-azacytidine (5-azaC) (Khoddami and Cairns, 2013, 2014). TET-assisted peroxotungstate oxidation sequencing (TAWO-seq) is a method that uses 5-azaC or peroxy chemically-dependent sequencing (Yuan et al., 2019). Nanopore sequencing is also capable of providing high-throughput sequencing of m5C (Garalde et al., 2018; Rang et al., 2018). Nanopore sequencing is also capable of providing high-throughput sequencing of m5C.

### 4.2 5-methylcytosine enzymes

m5C modifications are distributed in mRNA, enriched near the 5′UTR and 3′UTR, and also present in tRNA and rRNA (Trixl and Lusser, 2019). Several enzymes are capable of dynamically regulating m5C levels, including members of the NOP2/Sun RNA methyltransferase family (NSUNs), DNA methyltransferase family (DNMTs), Aly/REF export factor (ALYREF), tRNA aspartic acid methyltransferase family (TRDMTs), and AlkB homolog one histone H2A dioxygenase (ALKBH1) and Y-box binding protein 1 (YBX1), and TET methylcytosine dioxygenase family (TETs) (Reid et al., 1999; Goll et al., 2006; Yang et al., 2017a; Chen et al., 2019a; Bohnsack et al., 2019; Chellamuthu and Gray, 2020). NSUN1、NNSUN4, and NSUN5 methylated rRNAs.NSUN2, DNMT2, NSUN3, and NSUN6 methylated cytoplasmic tRNA, and NSUN2 also modifies ncRNA and mRNA. ALYREF, YBX1, and YTHDF2 can read m5C (Yang et al., 2017a). ALYREF is the most critical RNA m5C-binding protein known to participate in the mRNA exocytosis process in concert with NSUN2 by binding to m5C sites on mRNA. In eukaryotes, the most active m5C methyltransferases are DNMT2 and NSUN2 (Okamoto et al., 2012; Squires et al., 2012). NSUN2 m5C methylation of tRNA is associated with the survival, differentiation, and development of eukaryotes (Khan et al., 2012; Martinez et al., 2012; Blanco et al., 2014; Blanco et al., 2016). DNMT2 regulates tRNA differentiation, survival, and translation accuracy (Schaefer et al., 2010; Tuorto et al., 2012; Durdevic et al., 2013).

### 4.3 Role of 5-methylcytosine in hepatocellular carcinoma

m5C modifications have important implications for tumor progression and can act as an effective target for cancer treatment (He et al., 2020b; Nombela et al., 2021). The expression of m5C in HCC tissues was significantly higher than that in adjacent tissues (He et al., 2020a). m5C modifications and their regulators can regulate HCC cell proliferation, migration, and invasion, regulating HCC development and progression (Figure 3). It has been shown that m5C expression levels and regulators *NSUN4*, *NSUN5*, *DNMT1*, *TET2*, and *ALYREF* are significantly upregulated in HCC and are related to poor prognosis in HCC patients (Blanco et al., 2016; He et al., 2020a; He et al., 2020b; He et al., 2020c; Du et al., 2020;Zhang et al., 2020e; Cui et al., 2021b; He et al., 2021; Liu et al., 2022). DNMT1 is an independent risk factor, highly expressed in HCC tissues and positively associated with many tumor microenvironments (TME) infiltrating immune cells, promoting the malignant progression of HCC and strongly associated with poor prognosis in HCC patients (Gu et al., 2021). Another study found that overexpression of *ALYREF* and upregulation of eukaryotic translation initiation factor 4A3 (*eIF4A3*) were related to poor prognosis in HCC patients (Xue et al., 2021). The expression of *NSUN2* is upregulated in HCC, which promotes tumor progression by increasing the expression level of H19 imprinted maternally expressed transcript (H19) and promoting the binding of H19 to G3BP stress granule assembly factor 1 (G3BP1) (Sun et al., 2020). Another study found that *NSUN2* overexpression increased the stability of Fizzy-related-1 (*FZR1*) mRNA and promoted the proliferation of HCC cells (Zhai et al., 2021). These findings indicate that m5C-related regulatory genes, such as *NSUN2*, *NSUN4*, and *ALYREF*, can be used as potential biomarkers for HCC diagnosis.

## 5 N1-methyladenosine

First discovered in 1961, m1A is widely distributed in bacteria, archaea, and eukaryotes and is evolutionarily conserved (Figure 1D) (Dunn, 1961; Dominissini et al., 2016; Shi et al., 2020a). m1A is a methylation modification present in tRNA, mRNA, rRNA, and lncRNA, and mitochondrial RNA that is positively charged under physiological conditions. It can alter RNA structure, disrupt base-pairing specificity, affect the tertiary structure of ribosomes, and also regulates gene expression, controls cell fate, and influences disease onset and progression (Figure 2) (Agris, 1996; Delatte et al., 2016; Liu et al., 2016; Li et al., 2017a; Bhan et al., 2017; Oerum et al., 2017; Zhao et al., 2019b).

### 5.1 N1-methyladenosine high-throughput sequencing methods

m1A sequencing has enabled a comprehensive exploration of m1A methylation modifications in the transcriptome, providing an important tool for exploring m1A methylation function. m1A blocks Watson-Crick pairing, effectively blocking RT and inducing truncation or mutation of RT products (Yang et al., 2020). Based on the properties of m1A, the commonly used m1A high-throughput sequencing methods include m1A-seq-TGIRT、m1A-MAP、m1A-IP-seq, and m1A-quant-seq (Safra et al., 2017b; Dai et al., 2018; Zhou et al., 2019a). These methods provide the basis for in-depth studies of m1A and can contribute to a detailed exploration of the biological role of m1A.

### 5.2 N1-methyladenosine enzymes

m1A occurs in mRNA, tRNA, rRNA, and mitochondrial RNA transcripts. m1A modification has been extensively studied and is regulated by enzymes, including tRNA methyltransferases (TRMT6, TRMT10C, TRMT61A, and TRMT61B), readers (YTHDF1, YTHDF2, YTHDF3, and YTHDC1) and demethylases (ALKBH1, ALKBH3) (Dominissini et al., 2016; Dai et al., 2018; Scheitl et al., 2020; Esteve-Puig et al., 2021; Shafik et al., 2021). TRMT6 and TRMT61A are responsible for cytoplasmic tRNA and some nuclear mRNA m1A methylation with GUUCRA tRNA-like motifs. Underexpression of *TRMT6* and *TRMT61* reduces proliferation and death of C6 glioma cells, while their overexpression biological processes encode mRNAs involved in tumorigenic processes (Macari et al., 2016). The m1A modification at tRNA position 58 (m1A58) is conserved in many organisms (Hamdane et al., 2014). m1A58 modification mediated by TRMT6/61A has important roles in the replication of retroviral HIV-1 *in vivo* and tRNA modification (Fukuda et al., 2021). ALKB protein family members ALKBH1 and ALKBH3 are demethylases of m1A (Chujo and Suzuki, 2012). The YTH structural domain-containing proteins YTHDF1, YTHDF2, YTHDF3, and YTHDC1 are readers of m1A in RNA and can interact directly with m1A-containing RNA (Seo and Kleiner, 2020). YTDHF proteins recognize and bind multiple m1A-modified sequence motifs, demonstrating m1A destabilization of RNA transcripts and demonstrating its function in post-transcriptional gene regulation.

### 5.3 Role of N1-methyladenosine in hepatocellular carcinoma

The contribution of m1A modifications to the epigenetic regulation of gene expression during HCC development and progression has been progressively demonstrated. m1A methylation modifications and regulators can effectively regulate HCC progression (Figure 3). Using bioinformatics analysis found that m1A-related regulatory genes are altered in HCC and affect clinicopathological features and prognosis (Wang et al., 2019; Shi et al., 2020b). TRMT6, TRMT10C, and YTHDF1 are expressed at higher levels in HCC than in normal tissues and are associated with poor prognosis in HCC and could be used as prognostic biomarkers in HCC (Wang et al., 2019; Shi et al., 2020b). The MYC pathway and PI3K/AKT signaling pathway could be engaged in controlling m1A in HCC cells (Shi et al., 2020b). TRMT6/TRMT61A increases m 1 A methylation in tRNA subsets to increase peroxisome proliferator-activated receptor-delta (PPARδ) translation, which in turn triggers cholesterol synthesis to activate Hedgehog signaling. Therefore, the TRMT6/TRMT61A complex can be a potential target for HCC to provide a more effective treatment strategy for HCC patients (Wang et al., 2021b). Another study showed that *YTHDF1*, *YTHDF2*, *YTHDF3*, and *ALKBH3* expression levels were upregulated in HCC using a raw letter analysis and were closely associated with HCC metabolism, providing new insights into m1A modification and metabolic heterogeneity in HCC (Tong et al., 2022). These studies demonstrate that m1A RNA epigenetic modifications take a crucial part in regulating HCC development.

## 6 N3-methylcytosine

m3C is less abundant and is primarily found in eukaryotic tRNA and mRNA, and some rRNA (Figures 1E, 2) (Hall, 1963; Clark et al., 2016). m3C modifications are highly conserved and specific modifications that affect tRNA structure, ribosome binding affinity, decoding activity, and maintain anticodon folding and pairing functions, playing a key role in many biological processes (Auffinger and Westhof, 2001; Olejniczak and Uhlenbeck, 2006; D'Silva et al., 2011; Han et al., 2017; Li et al., 2022).

### 6.1 N3-methylcytosine high-throughput sequencing methods

The m3C modification significantly destabilizes Watson-Crick C: G pairing. High-throughput detection methods for m3C based on this feature include ALKB-facilitated RNA methylation sequencing (ARM-seq)、AlkAniline-seq and Hydrazine-aniline cleavage sequencing (HAC-seq) (Cozen et al., 2015; Marchand et al., 2018; Cui et al., 2021a; Marchand et al., 2021). These methods provide a research basis for exploring the biochemical and biomedical potential of m3C.

### 6.2 N3-methylcytosine enzymes

The four known m3C modifying enzymes, methyltransferase methylcytidine 2A (METTL2A), 2B (METTL2B), 6 (METTL6), and 8 (METTL8), all contain conserved S-adenosylmethionine (SAM) binding domains. ALKBH1 and ALKBH3 demethylate m3C in tRNAs, affecting RNA stability and preventing degradation (Ougland et al., 2004; Ueda et al., 2017). METTL2 and METTL6 regulate m3C in specific tRNAs to regulate m3C. METTL8 regulates m3C in mRNAs (Xu et al., 2017) and has recently been shown to be a mitochondrial tRNA m3C32 methyltransferase (Arimbasseri et al., 2016; Schöller et al., 2021). The formation of m3C in human mt-tRNAs depends on METTL8 (Towns and Begley, 2012; Suzuki et al., 2020; Schöller et al., 2021; Lentini et al., 2022), which can promote tumorigenesis by affecting genetic organization within or close to the nucleolus and regulatory R-loop formation through its m3C methyltransferase activity (Zhang et al., 2020d). ALKBH1 can demethylate m3C in mammalian cell mRNA and tRNA, providing m3C a single-stranded substrate preference in RNA and DNA and promoting proliferation, migration, and invasion of cancer cells (Aas et al., 2003; Sundheim et al., 2006; Chen et al., 2019d). Expression of *ALKBH3* is associated with tumor development and modification of protein synthesis, indicating that m3C may play a prominent part in cancer biology (Chen et al., 2019d).

### 6.3 Role of N3-methylcytosine in hepatocellular carcinoma

Studies have shown that m3C and its regulators play an important role in HCC (Figure 3). Bioinformatic analysis of METTL6 in HCC revealed that *METTL6* mRNA expression levels were significantly upregulated in HCC and were closely associated with poorer patient survival outcomes. In contrast, *METTL6* downregulation inhibited HCC progression by suppressing cell adhesion molecules and could be a potential therapeutic target for HCC (Bolatkan et al., 2022). METTL6-mediated m3C tRNA methylation regulates gene expression, cellular homeostasis, translation, and tumor cell growth (Ignatova et al., 2020; Bolatkan et al., 2022). Downregulation of METTL6 attenuates cell proliferation, invasion, and adhesion in HCC by inhibiting cell adhesion molecules and suppresses the malignant phenotype of HCC (Bolatkan et al., 2022). METTL6 deficiency decreases the metabolic level of hepatic cells, inhibits HCC proliferation, and is significantly associated with a low survival rate in patients with HCC (Ignatova et al., 2020). Another research indicated that m3C levels in mRNA in HCC tissues were remarkably decreased compared to normal tumor-adjacent tissues, potentially due to increased *ALKBH1* expression and decreased *METTL8* expression in HCC tissues (Ma et al., 2019).

## 7 Pseudouridine

Ψ is a five-carbon isomer of uridine, that is the first post-transcriptional modification to be identified, known as the “fifth nucleoside” of RNA, and found in tRNA, rRNA, snRNA, small nucleolar RNA (snoRNA), telomerase RNA, lncRNA, and polyadenylated mRNA (Figures 1F, 2) (Cohn, 1951, 1960; Fedorov and Bogomazov, 1969; Becker et al., 1998; Kim et al., 2010; Machnicka et al., 2013; Schwartz et al., 2014a; Carlile et al., 2014; Li et al., 2015). When bound to RNA, Ψ could change the structure of RNA, and improving base pairing, act in RNA folding, secondary structure, stability, and translation, and is regulated dynamically by the environment, which adds a potential mechanism for regulating RNA fate (Arnez and Steitz, 1994; Davis, 1995; Yu et al., 1998; Yarian et al., 1999; Zebarjadian et al., 1999; Newby and Greenbaum, 2001; Lecointe et al., 2002; Newby and Greenbaum, 2002; Nobles et al., 2002; King et al., 2003; Cabello-Villegas and Nikonowicz, 2005; Karikó et al., 2008; Bilbille et al., 2009; Karijolich and Yu, 2011; Fernández et al., 2013; Gu et al., 2013; Zhao and He, 2015; Hoernes et al., 2016; Safra et al., 2017a; Sloan et al., 2017; Bohnsack and Sloan, 2018; Zhao et al., 2018b; Guzzi et al., 2018; Penzo and Montanaro, 2018; Eyler et al., 2019).

### 7.1 Pseudouridine high-throughput sequencing methods

As research has progressed, several methods for Ψ have been developed. Mainly include Ψ-seq、PSI-seq、Pseudo-seq、CeU-Seq、HydraPsi-Seq and Nanopore RNA sequencing (Schwartz et al., 2014a; Carlile et al., 2014; Lovejoy et al., 2014; Li et al., 2015; Marchand et al., 2020; Begik et al., 2021; Thomas et al., 2021). These methods provide a research basis for exploring the biological role of Ψ.

### 7.2 Pseudouridine enzymes

Ψ is the most abundant type of post-transcriptional modification in ncRNA. An essential difference between pseudouridylation and methylation is that pseudouridylation occurs as an irreversible modification in mammals (Rasmuson and Björk, 1995; Sibert and Patton, 2012). The formation of pseudouridine is promoted by Ψ synthase (PUS or Ψ synthase), which is responsible for catalyzing Ψ in various RNA substrates, including mRNA, tRNA, rRNA, 5S RNA, snRNA, and snoRNA. Pseudouridylation can be achieved by two different and independent mechanisms, RNA-independent and RNA-dependent pseudouridylation. Pseudouridine obeys an RNA-independent mechanism in bacteria and humans, and an RNA-dependent mechanism in eukaryotes and archaea (Rintala-Dempsey and Kothe, 2017). RNA-independent pseudouridylation is catalyzed by PUS, which simultaneously performs substrate recognition and catalyzes uridine isomerization to Ψ in the absence of the RNA template strand, primarily modifying tRNA, rRNA, and snRNA (Becker et al., 1997a; Becker et al., 1997b; Hamma and Ferré-D'Amaré, 2006; Hur et al., 2006; Roovers et al., 2006; Rintala-Dempsey and Kothe, 2017). The RNA-dependent mechanism is mediated by the RNA-protein complex family of cassette H/ACA small ribonucleoproteins (snoRNPs), which consists of dyskerin Ψ synthase 1 (DKC1) and ribonucleoproteins NHP2, NOP10, and GAR1.

### 7.3 Role of pseudouridine in hepatocellular carcinoma

Ψ was the first RNA modification shown to be associated with cancer, and Ψ modifications and regulators have been proved to take an important part in HCC (Figure 3) (Zhao et al., 2004; Jana et al., 2017; Sperling et al., 2017; Ji et al., 2020). The study demonstrates serum Ψ concentration was increased in HCC patients (Amuro et al., 1988; Tamura et al., 1988). Studies have shown that HCC cells lacking snoRNA H/ACA box 24 (SNORA24)-guided Ψ modification has increased translation error coding and stop codon read-through frequencies and are associated with poor patient survival (McMahon et al., 2019). Another study used bioinformatics to analyze the expression and role of the H/ACA snoRNP gene family in HCC. The expression of the H/ACA snoRNP gene family was higher in HCC tissues than in normal or adjacent tissues, and their differential expression was strongly associated with poor prognosis and multiple immune cell infiltration in HCC patients and could be considered as biomarkers for the treatment of HCC(Zhang et al., 2021b). It has also been shown that overexpression of *DKC1* can serve as a marker of the proliferative potential of HCC cells and is an unfavorable prognostic factor for HCC (Liu et al., 2012). One study found that oxidatively modified cytoplasmic protein disulfide bond isomerase 3 (PDIA3) in HCC promoted DKC1-mediated survival of HCC and that DKC1 is a worthwhile target for HCC prediction and treatment (Ko et al., 2018). Except for DKC1, Ψ 5′-phosphatase (PUDP) has been identified as a potential oncogene for HCC (Yu et al., 2022). PUDP positively correlates with tumor immune cell infiltration, and immune checkpoint expression and causes poor prognosis and a poor response to immunotherapy in HCC patients (Yu et al., 2022). These studies suggest that Ψ and its modulators can be a potential biomarker and treatment target in HCC treatment.

Currently, aberrant post-transcriptional modifications in cancer cells are gradually being studied, and there are many assays to detect aberrant RNA epigenetic modifications in tumors (Table 1). Studies on RNA modifications in HCC have mainly used Quantitative Real-time PCR (qPCR) and western blot to detect the expression levels of major regulators of RNA modifications (Chen et al., 2018; Qiao et al., 2021). Northern blotting (Northern blot) was used to verify the trend of RNA expression levels (Chen et al., 2021e). High-throughput sequencing techniques such as RNA-seq or methylated RNA Immunoprecipitation (meRIP) were used to detect the expression levels of RNA modifications (Chen et al., 2018; Chen et al., 2019c; Lan et al., 2019; Lin et al., 2020b; Liu et al., 2020b; Chen et al., 2020; Wu et al., 2021a; Xia et al., 2021). Liquid chromatography-tandem mass spectrometry (LC-MS) was used to detect the expression levels of RNA modifications (Chen et al., 2021e; Qiao et al., 2021; Xia et al., 2021). For m6A modifications, some studies have used kits such as EpiQuik m6A RNA Methylation Quantification Kit to quantify the expression of m6A modifications based on absorbance or fluorescence intensity, and m6A-IP-qPCR to quantify the enriched RNA (Chen et al., 2019c; Liu et al., 2020b; Chen et al., 2020; Li et al., 2021d; Yang et al., 2021). For m7G modification and m5C modification, the study examined the expression levels of both RNA modifications using tRNA m7G reduction and cleavage sequencing (TRAC-seq) and bisulfite pyrophosphate sequencing, respectively (Sun et al., 2020). After determining the differential expression of RNA modifications in tumors, most of them were detected using RNA Binding Protein Immunoprecipitation Assay (RIP) technique, Co-Immunoprecipitation (Co-IP), and Chromatin Isolation by RNA Purification (CHIRP-Seq) and other methods to study the regulatory network of post-transcriptional modifications in tumors (Chen et al., 2019c; Lan et al., 2019; Wang et al., 2020a; Lin et al., 2020b; Chen et al., 2020; Sun et al., 2020; Wu et al., 2021a; Bo et al., 2021; Xia et al., 2021). These technical tools can confirm the interactions with RNA modifications and related proteins, and search for upstream or downstream genes that regulate tumor progression (Chen et al., 2018). The use of these assays can provide an in-depth study of the relationship between the abnormal expression of tumor-associated RNA modifications and tumor progression, investigate the mechanism of tumorigenesis, and lay the scientific foundation for tumor treatment.

**TABLE 1:** HCC cell lines and RNA modification assays.

Cell lines	Aberrant RNA modifications in HCC	Detection method	References
HepG2, Huh-7, MHCC97L	The expression levels of m6A and METTL3 are upregulated	Methylated RNA immunoprecipitation (MeRIP), RNA-sequencing (RNA-seq), m6A-Seq, qRT-PCR, western blot	Chen et al. (2018)
HepG2, MHCC-97H, SK-HEP-1-Luc	The expression levels of m6A and METTL3 are upregulated	LC-MS/MS, qRT-PCR, western blot	Qiao et al. (2021)
HepG2, MHCC97H, HEP3B, SMMC-7721	The expression levels of m6A and METTL3 are upregulated	m6A dot blot, qRT-PCR, western blot	Xu et al. (2020)
Hep3B, HCCLM3, MHCC97-L, HUH7	The expression levels of m6A and METTL3 are upregulated	EpiQuik m6A RNA Methylation Quantitative Kit, qRT-PCR, western blot	Li et al. (2021b)
SMMC-7721, Bel-7402, MHCC97, HepG2	The expression levels of m6A and METTL3 are upregulated	EpiQuik m6A RNA methylation quantification ELISA kit, m6A dot blot, qRT-PCR, western blot	Yang et al. (2021)
HepG-2, Hepa1-6, SMMC-7721, Bel-7402	Expression levels of m6A and METTL3 are downregulated in sorafenib-resistant HCC	MeRIP, m6A dot blot, m6A-RNA immunoprecipitation (RIP), qRT-PCR, western blot	Lin et al. (2020b)
Huh7, Hep3B	The expression levels of m6A and METTL3 are upregulated	RIP, RNA pull-down, MeRIP, qRT-PCR, western blot	Bo et al. (2021)
BEL-7404, HCCLM3, SK-Hep-1, SMMC-7721, MHCC-97H	The expression levels of m6A and METTL3 are upregulated	MeRIP, RNA-binding protein immunoprecipitation (RIP), qRT-PCR, western blot	Wu et al. (2021b)
Huh7, PLC/PRF/5, Hep3B, HCCLM3, MHCC97H, SMCC7721	The expression levels of m6A and WTAP are upregulated	RNA-seq, EpiQuik™ m6A RNA Methylation Quantification Kit (Colorimetric), m6A dot blot, MeRIP, Co-immunoprecipitation (Co-IP), qRT-PCR, western blot	Chen et al. (2019d)
SMMC-7721, BEL-7402, BEL-7404	The expression levels of m6A and WTAP are upregulated	High-through Sequencing, MeRIP-qPCR, qRT-PCR,	Li et al. (2021a)
Huh-7, Hep3B, HepG2, SK-Hep1, HCCLM3, SNU-182, SNU-449	The expression levels of m6A and KIAA1429 are upregulated	RNA-seq, MeRIP-seq, RIP, qRT-PCR, western blot	Lan et al. (2019)
Bel-7402, SMMC-7721, Bel-7404, HepG2, Huh-7	The expression levels of m6A and circ_KIAA1429 are upregulated	RIP, qRT-PCR, western blot	Wang et al. (2021e)
Hep3B, HepG2, Huh7, SMMC-7721, BEL-7402, BEL-7404, MHCC97H, MHCC97L, MHCC-LM, QGY7703	The expression level of m6A is upregulated and the expression level of FTO is downregulated	MeRIP, RNA-seq, m6A dot blot, m6A-IP-qPCR, qRT-PCR, western blot	Liu et al. (2020e)
Huh7, MHCC97H, HCCLM3, HepG2, Hep3B, PLC/PRF/5, SMCC7721, BEL7402	The expression level of m6A is upregulated and the expression level of ALKBH5 is downregulated	m6A dot blot, MeRIP-seq, RIP, MeRIP-qPCR, western blot	Chen et al. (2020)
Huh-7, Li-7, HCC-LM3, SUN-182, Hep-3B, Hep-G2	The expression levels of m7G and WDR4 are upregulated	Chromatin immunoprecipitation (ChIP), RNA-seq, dot blot, MeRIP, LC-MS/MS, co-IP, RIP	Xia et al. (2021)
Huh7, SNU-449, Hep3B, PLC/PRF/5, SK-Hep-1	The expression levels of m7G 、METTL1 and WDR4 are upregulated	qRT-PCR, Northern blot, western blot, coimmunoprecipitation, LC-MS, tRNA m7G reduction and cleavage sequencing (TRAC-seq), m7G methylated tRNA immunoprecipitation qPCR	Chen et al. (2021e)
HepG2	The expression levels of m5C and NSUN2 are upregulated	Bisulfite-PCR pyrosequencing, Chromatin isolation by RNA purification and mass spectrometry analysis (ChIRP-MS), RIP	Sun et al. (2020)

With the advancement of gene editing technology, gene knockdown of tumor-related genes using gene editing systems such as CRISPR/Cas9 has been widely studied (Doyle et al., 2012; Wu et al., 2019a; Brommage et al., 2019). Currently, studies have been conducted to target RNA-modified enzymes for gene knockout and prepare knockout mouse models for studying the treatment of diseases such as tumors. One study used a lentivirus-based clustered regularly interspaced short palindromic repeat (CRISPR) gene editing system (lentiCRISPR v2) to knock out METTL3 in the HCC cell line Huh-7, and subsequently injected the knocked-out METTL3 cell line *in situ* into the left liver lobe of nude mice using *in situ* transplantation experiments, and found that knocking out METTL3 significantly inhibited the growth of HCC tumors *in situ* in the liver of nude mice (Chen et al., 2018). Another study using CRISPR/Cas9 technology to construct liver-specific METTL1 knockout mice showed a significant reduction in HCC tumor lesions and a significant decrease in tumor load (Chen et al., 2021e). In the study of HCC, some studies have used gene editing technology to construct knockout mouse models to knock out RNA-modified regulators, which can more scientifically validate the role of regulators in HCC (Chen et al., 2018; Li et al., 2021c; Chen et al., 2021e). These animal models have played an important role in studying the mechanism of action of m6A in HCC.

## 8 Discussion

RNA modifications play a key role in regulating cell fate, an important regulator of HCC, in tumorigenesis and disease progression (Han et al., 2018; Pea et al., 2021; Braghini et al., 2022). The m6A, m7G, m5C, m1A, m3C, and ψ RNA modifications dynamically regulate the development of HCC, providing new strategies and possibilities for selecting possible therapeutic targets and investigating precisely directed intervention strategies for future HCC therapy (Tian et al., 2019; Bolatkan et al., 2022; Liu et al., 2022; Ren et al., 2022; Tong et al., 2022; Yu et al., 2022). As the most intensively studied RNA modification, m6A, and its regulators regulate the proliferation, migration, invasion, and EMT processes of HCC cells and are promising targets for HCC therapy (Zhou et al., 2019b; Liu et al., 2020a; Wang et al., 2020b; Zhang et al., 2020c; Qu et al., 2020; Wu et al., 2020; Zhu et al., 2020; Li et al., 2021e; Wei, 2021; Gu et al., 2022; Ren et al., 2022). Although m6A in mammalian RNA has been extensively studied, there is currently no evidence for DNA N6 -methyladenine in mammals (Douvlataniotis et al., 2020). It suggests that m6A may regulate mammalian life activities only through modification of RNA, and that regulation of m6A modification in RNA can effectively mitigate the progression of diseases such as HCC. m7G modifications and their regulators play an integral part in the growth, and invasion of HCC cells and are associated with poor patient prognosis and chemotherapy resistance, representing valuable markers for clinical diagnosis and poor prognosis (Dai et al., 2021). m5C modifications and their regulators can regulate HCC cells (Xue et al., 2021). m1A, m3C, and Ψ methylation modifications and their regulators can also effectively regulate HCC progression (McMahon et al., 2019; Shi et al., 2020b; Bolatkan et al., 2022). The differential expression of RNA modifications in HCC can be quantified by RNA technologies to investigate their precise biological role in HCC occurrence and progression (Zheng et al., 2020; Cao et al., 2021). With advances in sequencing technology, increasing numbers of RNA modifications can be localized and quantified at a single nucleotide resolution (Edelheit et al., 2013; Schwartz et al., 2014a; Cozen et al., 2015; Linder et al., 2015; Li et al., 2017a; Zheng et al., 2020; Cao et al., 2021). Existing sequencing technologies cannot achieve 100% accuracy and sensitivity, and there is no suitable sequencing technology to detect several RNA modifications in the same transcript simultaneously (Wang et al., 2021c). The most advanced sequencing technology available is nanopore sequencing, extending the scope of RNA modification studies, but it still has limitations (Workman et al., 2019; Pratanwanich et al., 2021; Wang et al., 2021e). Therefore, the development of technologies for identifying and quantifying RNA modifications is critical for advancing understanding of their role in cancer development processes in HCC. Although unusual expression of RNA modifying enzymes has now been described in the development of HCC, further investigation is required for the exact contribution of these enzymes and the corresponding modifications to HCC metastasis, and resistance requires further investigation.

## 9 Conclusion

In conclusion, although RNA modifications and differential expression of associated regulatory genes have been explored in most physiological processes of HCC and development, their precise role and effects on tumorigenesis, proliferation, metastasis, and resistance still require further investigation. In this review, we have summarized existing knowledge on the important roles and regulatory mechanisms of m6A, m7G, m5C, m1A, m3C, and ψ in HCC, suggesting that targeting aberrant post-transcriptional modifications in cancer cells has the potential to be an effective tool for HCC treatment.
