# Supplementary material for: The role of RNA modification in hepatocellular carcinoma
Source: Front Pharmacol. 2022 Sep 2;13:984453. doi: 10.3389/fphar.2022.984453 (PMC9479111; doi:10.3389/fphar.2022.984453)
Supplement: Supplementary file 1 [file Table1.docx]

Supplementary Material

Table S1.The role of RNA epigenetic modifications in HCC progression.

| Modification type | Regulator type | Regulators | Expression | Clinical characters | Function in HCC | Mechanism | Reference |
| --- | --- | --- | --- | --- | --- | --- | --- |
| m6A | Writer | METTL3 | Up | Poor prognosis | Promotes HCC cell proliferation, migration, and colony formation | METTL3 inhibits SOCS2 expression through an m6A-YTHDF2-dependent mechanism | (Chen et al., 2018) |
|  |  |  | Up | Poor prognosis | Promotes HCC cell growth, proliferation, and epithelial-mesenchymal transition | METTL3 promotes HCC via the UBC9/SUMOylated METTL3/Snail axis | (Xu et al., 2020) |
|  |  |  | Up | Poor prognosis | Participation in the formation of VMs in HCC | METTL3 and YAP1 promote angiogenic mimetic formation in HCC | (Qiao et al., 2021) |
|  |  |  | Up | Poor prognosis | Promotes HCC cell invasion, migration, and proliferation | METTL3 positively regulates the expression of USP7 | (Li et al., 2021c) |
|  |  |  | Up | Poor prognosis | Promotes glycolysis in HCC cells | METTL3 downregulation inhibits mTORC1 activity and synergizes with the glycolysis inhibitor 2-deoxyglucose | (Lin et al., 2020a) |
|  |  |  | Up | Poor prognosis | Promotes metabolic reprogramming of HCC cells | HBXIP mediates METTL3 to regulate the expression of HIF-1α | (Yang et al., 2021) |
|  |  |  | Down | Poor prognosis | Activation of autophagic signaling pathway and promotion of sorafenib resistance in HCC cells | METTL3/ FOXO3 axis regulates HCC autophagy and sorafenib resistance | (Lin et al., 2020b) |
|  |  |  | Down | Poor prognosis | Promotes HCC cell resistance to sorafenib | LINC01273/miR-600/METTL3 axis for sorafenib-resistant HCC | (Kong et al., 2022) |
|  |  |  | Up | Poor prognosis | Promotes proliferation, colony formation, migration, and invasion of HCC cells | METTL3 promotes HCC progression by regulating the NIFK-AS1/miR-637/AKT1 axis | (Chen et al., 2021b) |
|  |  |  | Up | Poor prognosis | Promote the proliferation of HCC cells | METTL3 regulates the MEG3/miR-544b/BTG2 axis to regulate HCC progression | (Wu et al., 2021b) |
|  |  |  | Up | Poor prognosis | Promote the proliferation of HCC cells | ILF3-AS1 regulates ILF3 expression through the recruitment of METTL3 to promote HCC progression | (Bo et al., 2021) |
|  |  |  | Up | Poor prognosis | Promote the proliferation of HCC cells | METTL3 mediates LINC00958 upregulation and promotes HCC progression through miR-3619-5p/HDGF axis | (Zuo et al., 2020) |
|  |  |  | Up | Poor prognosis | Promote the proliferation of HCC cells | METTL3 upregulates MAAS expression and thus promotes HBV+HCC progression | (Tao et al., 2022) |
|  |  |  | Up | Poor prognosis | Promote the proliferation of HCC cells | METTL3 regulates the circ-ARL3/miR-1305 axis to promote the progression of HBV+HCC | (Rao et al., 2021) |
|  |  |  | Up | Poor prognosis | Promote the proliferation of HCC cells | METTL3 mediates the hsa_circ_0058493-YTHDC1 axis to promote HCC progression | (Wu et al., 2021a) |
|  |  |  | Up | Poor prognosis | Promote the proliferation of HCC cells | METTL3 mediates the circHPS5/ miR-370/HMGA2 axis to promote the development of HCC | (Rong et al., 2021) |
|  |  | METTL14 | Down | Good prognosis | Inhibits migration, invasion, and metastasis of HCC cells | METTL14 interacts with DGCR8 to regulate microRNA 126 to suppress HCC progression | (Ma et al., 2017) |
|  |  |  | Down | Good prognosis | Inhibition of migration, invasion, and EMT of HCC cells | METTL14 regulates the EGFR/PI3K/AKT signaling pathway | (Shi et al., 2020) |
|  |  |  | Down | Good prognosis | Inhibits migration and invasion of HCC cells | METTL14 mediates HNF3γ regulation of HCC cell differentiation | (Zhou et al., 2020) |
|  |  |  | Down | Good prognosis | Inhibits migration and invasion of HCC cells | Progress of HIF-1α/METTL14/YTHDF2/SLC7A11 axis in regulating hypoxic HCC | (Fan et al., 2021b) |
|  |  |  | Down | Good prognosis | Inhibits migration and invasion of HCC cells | METTL14-USP48-SIRT6 axis regulates HCC metabolism by modulating glycolysis | (Du et al., 2021) |
|  |  | WTAP | Up | Poor prognosis | Inhibits migration and invasion of HCC cells | WTAP promotes HCC progression via HuR-ETS1-p21/p27 axis | (Chen et al., 2019) |
|  |  | ZC3H13 | Down | Good prognosis | Inhibits migration and invasion of HCC cells | The miR-362-3p/miR-425-5p-ZC3H13 axis regulates HCC progression | (Wu et al., 2022) |
|  |  | KIAA1429 | Up | Poor prognosis | Promote the proliferation and metastasis of HCC cells | KIAA1429 mediates GATA3 expression to promote HCC progression | (Lan et al., 2019) |
|  |  |  | Up | Poor prognosis | Promotes proliferation, migration, and invasion of HCC cells | KIAA1429 inhibits ID2 to promote the progression of HCC | (Cheng et al., 2019) |
|  |  |  | Up | Poor prognosis | Promotes migration, invasion, and EMT of HCC | circ_KIAA1429 mediates the m6A-YTHDF3-Zeb1 axis to accelerate the progression of HCC | (Wang et al., 2020) |
|  |  |  | Up | Poor prognosis | Promotes proliferation, migration, and invasion of HCC cells | KIAA1429 regulates HCC progression via the circDLC1-HuR-MMP1 axis | (Liu et al., 2021a) |
|  |  | METTL16 | Up | Poor prognosis | Promotes proliferation, migration, and invasion of HCC cells | METTL16 promotes HCC progression by regulating the expression of eIF3b | (Su et al., 2022) |
|  | Eraser | FTO | Up | Poor prognosis | Promotes proliferation, migration, and invasion of HCC cells | Depletion of FTO by SIRT1 down-regulates GNAO1 expression | (Liu et al., 2020a) |
|  |  |  | Up | Poor prognosis | Promote the proliferation of HCC cells | FTO reduces CUL4A abundance to inhibit HCC progression | (Mittenbühler et al., 2020) |
|  |  |  | Up | Poor prognosis | Promote the proliferation of HCC cells | FTO promotes HCC development by mediating PKM2 demethylation | (Li et al., 2019) |
|  |  |  | Up | Poor prognosis | Promote the proliferation of HCC cells | SNPs of FTO have prognostic value in HCC patients treated with TACE | (Liu et al., 2021b) |
|  |  | ALKBH5 | Down | Good prognosis | Inhibit the proliferation and invasion of HCC cells | Dysregulation of the ALKBH5/LYPD1 axis drives the progression of HCC | (Chen et al., 2020) |
|  |  |  | Up | Poor prognosis | Promoting the progress of HBV-HCC | Depletion of ALKBH5 inhibits the progression of HBV-HCC | (Qu et al., 2021) |
|  |  |  | Up | Poor prognosis | Promotes proliferation, migration, and invasion of HCC cells | ALKBH5 mediates the NEAT1/miR-214 axis to promote the progression of HCC | (Yeermaike et al., 2022) |
|  |  |  | Up | Poor prognosis | Promotes proliferation, migration, and invasion of HCC cells | ALKBH5 mediates abnormal activation of the circCPSF6-YAP1 axis to promote the malignant development of HCC | (Chen et al., 2022) |
|  | Reader | YTHDF1 | Up | Poor prognosis | Promotes proliferation, migration, and invasion of HCC cells | YTHDF1-mediated FZD5 expression regulates the WNT/β-catenin axis | (Liu et al., 2020b) |
|  |  |  | Up | Poor prognosis | Promotes proliferation, migration, and invasion of HCC cells | YTHDF1 promotes HCC progression by promoting EMT and activating AKT/GSK3β/β-catenin signaling | (Bian et al., 2020) |
|  |  |  | Up | Poor prognosis | Promote proliferation, migration, invasion, and EMT of HCC cells | YTHDF1 induces EMT through activation of PI3K/AKT/mTOR signaling pathway to promote HCC progression | (Luo et al., 2021) |
|  |  |  | Up | Poor prognosis | Regulation of immune cell infiltration in HCC | YTHDF1 regulates CD3+ and CD8+ T cell infiltration | (Li et al., 2021a) |
|  |  |  | Up | Poor prognosis | Driving hypoxia-induced autophagy | HIF-1α-induced YTHDF1 expression drives hypoxia-induced autophagy in HCC by promoting ATG2A and ATG14 translation | (Li et al., 2021b) |
|  |  |  | Up | Poor prognosis | Promote the proliferation and metastasis of HCC cells | The m6A-YTHDF1-EGFR axis promotes the progression of HCC after IRFA | (Su et al., 2021) |
|  |  |  | Up | Poor prognosis | Promotes proliferation, migration, and invasion of HCC cells | CircMAP2K4/miR-139-5p/YTHDF1 axis promotes HCC progression | (Chi et al., 2021) |
|  |  |  | Up | Poor prognosis | Promotes proliferation, migration, and invasion of HCC cells | The circRHBDD1/YTHDF1/PIK3R1 axis regulates the metabolism of HCC | (Cai et al., 2022) |
|  |  | YTHDF2 | Up | Poor prognosis | Promotes proliferation, migration, and invasion of HCC cells | YTHDF2 promotes HCC progression by regulating OCT4 | (Zhang et al., 2020) |
|  |  |  | Up | Poor prognosis | Promotes proliferation, migration, and invasion of HCC cells | miR-145 regulates HCC progression by targeting YTHDF2 | (Yang et al., 2017) |
|  |  |  | Down | Good prognosis | Inhibits proliferation, migration, and invasion of HCC cells | YTHDF2 inhibits HCC cell proliferation and growth by disrupting EGFR mRNA in HCC under hypoxic conditions | (Zhong et al., 2019) |
|  |  |  | Down | Good prognosis | Inhibits the proliferation of HCC cells and reduces vascular density and permeability | YTHDF2 regulates the decay of IL11, SERPINE2, and vascular abnormalities in HCC | (Hou et al., 2019) |
|  |  |  | Down | Good prognosis | Inhibits proliferation, migration, and invasion of HCC cells | YTHDF2 mediates the lncAY/BMI1/Wnt/β-catenin axis | (Chen et al., 2021a) |
|  |  | YTHDF3 | Up | Poor prognosis | Promotes proliferation, migration, and invasion of HCC cells | KDM5B promotes HCC progression via miR-448/YTHDF3/ITGA6 axis | (Guo et al., 2021) |
|  |  | IGF2BP1 | Up | Poor prognosis | Promotes proliferation, migration, and invasion of HCC cells | RBM15 mediates the YES1-MAPK axis to promote HCC progression through an IGF2BP1-dependent manner | (Cai et al., 2021) |
|  |  |  | Up | Poor prognosis | Promotes proliferation, migration, and invasion of HCC cells | METTL3/IGF2BP1/CD47-mediated EMT transition contributes to incomplete ablation-induced metastasis of HCC cells | (Fan et al., 2021a) |
|  |  |  | Up | Poor prognosis | Promotes proliferation, migration, and invasion of HCC cells | IGF2BP1 stabilizes c-MYC and MKI67 mRNA and enhances c-Myc and Ki-67 protein translation | (Gutschner et al., 2014) |
|  |  |  | Up | Poor prognosis | Promotes proliferation, migration, and invasion of HCC cells | LINC01093-IGF2BP1-GLI1 Axis inhibits HCC progression | (He et al., 2019) |
|  |  |  | Up | Poor prognosis | Promotes proliferation, migration, and invasion of HCC cells | IGF2BP1 promotes HCC progression by facilitating circMAP3K4 translation | (Duan et al., 2022) |
|  |  | IGF2BP2 | Up | Poor prognosis | Promotes proliferation, migration, and invasion of HCC cells | IGF2BP2 promotes HCC progression through an m6A-FEN1-dependent mechanism | (Pu et al., 2020) |
|  |  | IGF2BP3 | Up | Poor prognosis | Promotes proliferation, migration, and invasion of HCC cells | Upregulation of IGF2BP3 expression by pgRNA promotes the progression of HCC | (Ding et al., 2021) |
| m7G | Writer | METTL1 | Up | Poor prognosis | Promotes proliferation, migration, and invasion of HCC cells | METTL1 promotes HCC progression through PTEN/AKT axis | (Tian et al., 2019) |
|  |  |  | Up | Poor prognosis | Promotes proliferation, migration, and invasion of HCC cells | METTL1 promotes HCC progression by mediating tRNA m7G modifications to promote the translation of target mRNAs | (Chen et al., 2021c) |
|  |  | WDR4 | Up | Poor prognosis | Promotes proliferation, migration, and invasion of HCC cells | WDR4 promotes proliferation, metastasis, and sorafenib resistance through induction of CCNB1 translation in HCC | (Xia et al., 2021) |
| m5C | Writer | ALYREF | Up | Poor prognosis | Promotes proliferation, migration, and invasion of HCC cells | ALYREF and eIF4A3 act synergistically to promote HCC progression | (Xue et al., 2021) |
|  |  | NSUN2 | Up | Poor prognosis | Promotes migration, invasion, and angiogenesis of HCC cells | NSUN2 promotes HCC progress through the MYC-NSUN2-H19-G3BP1 axis | (Sun et al., 2020) |
|  |  |  | Up | Poor prognosis | Promotes proliferation, migration, and invasion of HCC cells | NSUN2 overexpression mediates FZR1 to promote HCC progression | (Zhai et al., 2021) |
| m1A | Writer | TRMT6/TRMT61A | Up | Poor prognosis | Promotes proliferation, migration, and invasion of HCC cells | TRMT6/TRMT61A promotes HCC tumorigenesis by regulating PPARδ to promote cholesterol synthesis | (Wang et al., 2021) |
|  |  | METTL6 | Up | Poor prognosis | Promotes proliferation, migration, invasion, and adhesion of HCC cells | METTL6 downregulation inhibits HCC progression by suppressing cell adhesion molecules | (Bolatkan et al., 2022) |
|  |  |  | Up | Poor prognosis | Promotes proliferation, migration, and invasion of HCC cells | METTL6 deficiency reduces the metabolic level of the liver to inhibit the process of HCC | (Ignatova et al., 2020) |
|  |  | METTL8 | Up | Poor prognosis | Promotes proliferation, migration, and invasion of HCC cells | METTL8 mediates the expression of m3C in mRNA to promote HCC progression | (Ma et al., 2019) |
| Ψ | Writer | Dyskerin | Up | Poor prognosis | Promotes proliferation, migration and invasion of HCC cells | PDIA3 promotes DKC1-mediated HCC progression | (Ko et al., 2018) |
|  |  | PUDP | Up | Poor prognosis | Regulation of immune infiltration of HCC cells | PUDP affects HCC progression by interacting with immunosuppressive cells | (Yu et al., 2022) |

Bian, S., Ni, W., Zhu, M., Song, Q., Zhang, J., Ni, R., et al. (2020). Identification and Validation of the N6-Methyladenosine RNA Methylation Regulator YTHDF1 as a Novel Prognostic Marker and Potential Target for Hepatocellular Carcinoma. *Front Mol Biosci* 7**,** 604766. doi: 10.3389/fmolb.2020.604766.

Bo, C., Li, N., He, L., Zhang, S., and An, Y. (2021). Long non-coding RNA ILF3-AS1 facilitates hepatocellular carcinoma progression by stabilizing ILF3 mRNA in an m(6)A-dependent manner. *Hum Cell* 34(6)**,** 1843-1854. doi: 10.1007/s13577-021-00608-x.

Bolatkan, A., Asada, K., Kaneko, S., Suvarna, K., Ikawa, N., Machino, H., et al. (2022). Downregulation of METTL6 mitigates cell progression, migration, invasion and adhesion in hepatocellular carcinoma by inhibiting cell adhesion molecules. *Int J Oncol* 60(1). doi: 10.3892/ijo.2021.5294.

Cai, J., Chen, Z., Zhang, Y., Wang, J., Zhang, Z., Wu, J., et al. (2022). CircRHBDD1 augments metabolic rewiring and restricts immunotherapy efficacy via m(6)A modification in hepatocellular carcinoma. *Mol Ther Oncolytics* 24**,** 755-771. doi: 10.1016/j.omto.2022.02.021.

Cai, X., Chen, Y., Man, D., Yang, B., Feng, X., Zhang, D., et al. (2021). RBM15 promotes hepatocellular carcinoma progression by regulating N6-methyladenosine modification of YES1 mRNA in an IGF2BP1-dependent manner. *Cell Death Discov* 7(1)**,** 315. doi: 10.1038/s41420-021-00703-w.

Chen, M., Wei, L., Law, C.T., Tsang, F.H., Shen, J., Cheng, C.L., et al. (2018). RNA N6-methyladenosine methyltransferase-like 3 promotes liver cancer progression through YTHDF2-dependent posttranscriptional silencing of SOCS2. *Hepatology* 67(6)**,** 2254-2270. doi: 10.1002/hep.29683.

Chen, M.H., Fu, L.S., Zhang, F., Yang, Y., and Wu, X.Z. (2021a). LncAY controls BMI1 expression and activates BMI1/Wnt/β-catenin signaling axis in hepatocellular carcinoma. *Life Sci* 280**,** 119748. doi: 10.1016/j.lfs.2021.119748.

Chen, Y., Ling, Z., Cai, X., Xu, Y., Lv, Z., Man, D., et al. (2022). Activation of YAP1 by N6-Methyladenosine-Modified circCPSF6 Drives Malignancy in Hepatocellular Carcinoma. *Cancer Res* 82(4)**,** 599-614. doi: 10.1158/0008-5472.Can-21-1628.

Chen, Y., Peng, C., Chen, J., Chen, D., Yang, B., He, B., et al. (2019). WTAP facilitates progression of hepatocellular carcinoma via m6A-HuR-dependent epigenetic silencing of ETS1. *Mol Cancer* 18(1)**,** 127. doi: 10.1186/s12943-019-1053-8.

Chen, Y., Zhao, Y., Chen, J., Peng, C., Zhang, Y., Tong, R., et al. (2020). ALKBH5 suppresses malignancy of hepatocellular carcinoma via m(6)A-guided epigenetic inhibition of LYPD1. *Mol Cancer* 19(1)**,** 123. doi: 10.1186/s12943-020-01239-w.

Chen, Y.T., Xiang, D., Zhao, X.Y., and Chu, X.Y. (2021b). Upregulation of lncRNA NIFK-AS1 in hepatocellular carcinoma by m(6)A methylation promotes disease progression and sorafenib resistance. *Hum Cell* 34(6)**,** 1800-1811. doi: 10.1007/s13577-021-00587-z.

Chen, Z., Zhu, W., Zhu, S., Sun, K., Liao, J., Liu, H., et al. (2021c). METTL1 promotes hepatocarcinogenesis via m(7) G tRNA modification-dependent translation control. *Clin Transl Med* 11(12)**,** e661. doi: 10.1002/ctm2.661.

Cheng, X., Li, M., Rao, X., Zhang, W., Li, X., Wang, L., et al. (2019). KIAA1429 regulates the migration and invasion of hepatocellular carcinoma by altering m6A modification of ID2 mRNA. *Onco Targets Ther* 12**,** 3421-3428. doi: 10.2147/ott.S180954.

Chi, F., Cao, Y., and Chen, Y. (2021). Analysis and Validation of circRNA-miRNA Network in Regulating m(6)A RNA Methylation Modulators Reveals CircMAP2K4/miR-139-5p/YTHDF1 Axis Involving the Proliferation of Hepatocellular Carcinoma. *Front Oncol* 11**,** 560506. doi: 10.3389/fonc.2021.560506.

Ding, W.B., Wang, M.C., Yu, J., Huang, G., Sun, D.P., Liu, L., et al. (2021). HBV/Pregenomic RNA Increases the Stemness and Promotes the Development of HBV-Related HCC Through Reciprocal Regulation With Insulin-Like Growth Factor 2 mRNA-Binding Protein 3. *Hepatology* 74(3)**,** 1480-1495. doi: 10.1002/hep.31850.

Du, L., Li, Y., Kang, M., Feng, M., Ren, Y., Dai, H., et al. (2021). USP48 Is Upregulated by Mettl14 to Attenuate Hepatocellular Carcinoma via Regulating SIRT6 Stabilization. *Cancer Res* 81(14)**,** 3822-3834. doi: 10.1158/0008-5472.Can-20-4163.

Duan, J.L., Chen, W., Xie, J.J., Zhang, M.L., Nie, R.C., Liang, H., et al. (2022). A novel peptide encoded by N6-methyladenosine modified circMAP3K4 prevents apoptosis in hepatocellular carcinoma. *Mol Cancer* 21(1)**,** 93. doi: 10.1186/s12943-022-01537-5.

Fan, Z., Gao, Y., Zhang, W., Yang, G., Liu, P., Xu, L., et al. (2021a). METTL3/IGF2BP1/CD47 contributes to the sublethal heat treatment induced mesenchymal transition in HCC. *Biochem Biophys Res Commun* 546**,** 169-177. doi: 10.1016/j.bbrc.2021.01.085.

Fan, Z., Yang, G., Zhang, W., Liu, Q., Liu, G., Liu, P., et al. (2021b). Hypoxia blocks ferroptosis of hepatocellular carcinoma via suppression of METTL14 triggered YTHDF2-dependent silencing of SLC7A11. *J Cell Mol Med* 25(21)**,** 10197-10212. doi: 10.1111/jcmm.16957.

Guo, J.C., Liu, Z., Yang, Y.J., Guo, M., Zhang, J.Q., and Zheng, J.F. (2021). KDM5B promotes self-renewal of hepatocellular carcinoma cells through the microRNA-448-mediated YTHDF3/ITGA6 axis. *J Cell Mol Med* 25(13)**,** 5949-5962. doi: 10.1111/jcmm.16342.

Gutschner, T., Hämmerle, M., Pazaitis, N., Bley, N., Fiskin, E., Uckelmann, H., et al. (2014). Insulin-like growth factor 2 mRNA-binding protein 1 (IGF2BP1) is an important protumorigenic factor in hepatocellular carcinoma. *Hepatology* 59(5)**,** 1900-1911. doi: 10.1002/hep.26997.

He, J., Zuo, Q., Hu, B., Jin, H., Wang, C., Cheng, Z., et al. (2019). A novel, liver-specific long noncoding RNA LINC01093 suppresses HCC progression by interaction with IGF2BP1 to facilitate decay of GLI1 mRNA. *Cancer Lett* 450**,** 98-109. doi: 10.1016/j.canlet.2019.02.033.

Hou, J., Zhang, H., Liu, J., Zhao, Z., Wang, J., Lu, Z., et al. (2019). YTHDF2 reduction fuels inflammation and vascular abnormalization in hepatocellular carcinoma. *Mol Cancer* 18(1)**,** 163. doi: 10.1186/s12943-019-1082-3.

Ignatova, V.V., Kaiser, S., Ho, J.S.Y., Bing, X., Stolz, P., Tan, Y.X., et al. (2020). METTL6 is a tRNA m(3)C methyltransferase that regulates pluripotency and tumor cell growth. *Sci Adv* 6(35)**,** eaaz4551. doi: 10.1126/sciadv.aaz4551.

Ko, E., Kim, J.S., Ju, S., Seo, H.W., Chang, Y., Kang, J.A., et al. (2018). Oxidatively Modified Protein-Disulfide Isomerase-Associated 3 Promotes Dyskerin Pseudouridine Synthase 1-Mediated Malignancy and Survival of Hepatocellular Carcinoma Cells. *Hepatology* 68(5)**,** 1851-1864. doi: 10.1002/hep.30039.

Kong, H., Sun, J., Zhang, W., Zhang, H., and Li, H. (2022). Long intergenic non-protein coding RNA 1273 confers sorafenib resistance in hepatocellular carcinoma via regulation of methyltransferase 3. *Bioengineered* 13(2)**,** 3108-3121. doi: 10.1080/21655979.2022.2025701.

Lan, T., Li, H., Zhang, D., Xu, L., Liu, H., Hao, X., et al. (2019). KIAA1429 contributes to liver cancer progression through N6-methyladenosine-dependent post-transcriptional modification of GATA3. *Mol Cancer* 18(1)**,** 186. doi: 10.1186/s12943-019-1106-z.

Li, J., Wang, W., Zhou, Y., Liu, L., Zhang, G., Guan, K., et al. (2021a). m6A Regulator-Associated Modification Patterns and Immune Infiltration of the Tumor Microenvironment in Hepatocarcinoma. *Front Cell Dev Biol* 9**,** 687756. doi: 10.3389/fcell.2021.687756.

Li, J., Zhu, L., Shi, Y., Liu, J., Lin, L., and Chen, X. (2019). m6A demethylase FTO promotes hepatocellular carcinoma tumorigenesis via mediating PKM2 demethylation. *Am J Transl Res* 11(9)**,** 6084-6092.

Li, Q., Ni, Y., Zhang, L., Jiang, R., Xu, J., Yang, H., et al. (2021b). HIF-1α-induced expression of m6A reader YTHDF1 drives hypoxia-induced autophagy and malignancy of hepatocellular carcinoma by promoting ATG2A and ATG14 translation. *Signal Transduct Target Ther* 6(1)**,** 76. doi: 10.1038/s41392-020-00453-8.

Li, Y., Cheng, X., Chen, Y., Zhou, T., Li, D., and Zheng, W.V. (2021c). METTL3 facilitates the progression of hepatocellular carcinoma by modulating the m6A level of USP7. *Am J Transl Res* 13(12)**,** 13423-13437.

Lin, Y., Wei, X., Jian, Z., and Zhang, X. (2020a). METTL3 expression is associated with glycolysis metabolism and sensitivity to glycolytic stress in hepatocellular carcinoma. *Cancer Med* 9(8)**,** 2859-2867. doi: 10.1002/cam4.2918.

Lin, Z., Niu, Y., Wan, A., Chen, D., Liang, H., Chen, X., et al. (2020b). RNA m(6) A methylation regulates sorafenib resistance in liver cancer through FOXO3-mediated autophagy. *Embo j* 39(12)**,** e103181. doi: 10.15252/embj.2019103181.

Liu, H., Lan, T., Li, H., Xu, L., Chen, X., Liao, H., et al. (2021a). Circular RNA circDLC1 inhibits MMP1-mediated liver cancer progression via interaction with HuR. *Theranostics* 11(3)**,** 1396-1411. doi: 10.7150/thno.53227.

Liu, J., Wang, D., Zhou, J., Wang, L., Zhang, N., Zhou, L., et al. (2021b). N6-methyladenosine reader YTHDC2 and eraser FTO may determine hepatocellular carcinoma prognoses after transarterial chemoembolization. *Arch Toxicol* 95(5)**,** 1621-1629. doi: 10.1007/s00204-021-03021-3.

Liu, X., Liu, J., Xiao, W., Zeng, Q., Bo, H., Zhu, Y., et al. (2020a). SIRT1 Regulates N(6) -Methyladenosine RNA Modification in Hepatocarcinogenesis by Inducing RANBP2-Dependent FTO SUMOylation. *Hepatology* 72(6)**,** 2029-2050. doi: 10.1002/hep.31222.

Liu, X., Qin, J., Gao, T., Li, C., He, B., Pan, B., et al. (2020b). YTHDF1 Facilitates the Progression of Hepatocellular Carcinoma by Promoting FZD5 mRNA Translation in an m6A-Dependent Manner. *Mol Ther Nucleic Acids* 22**,** 750-765. doi: 10.1016/j.omtn.2020.09.036.

Luo, X., Cao, M., Gao, F., and He, X. (2021). YTHDF1 promotes hepatocellular carcinoma progression via activating PI3K/AKT/mTOR signaling pathway and inducing epithelial-mesenchymal transition. *Exp Hematol Oncol* 10(1)**,** 35. doi: 10.1186/s40164-021-00227-0.

Ma, C.J., Ding, J.H., Ye, T.T., Yuan, B.F., and Feng, Y.Q. (2019). AlkB Homologue 1 Demethylates N(3)-Methylcytidine in mRNA of Mammals. *ACS Chem Biol* 14(7)**,** 1418-1425. doi: 10.1021/acschembio.8b01001.

Ma, J.Z., Yang, F., Zhou, C.C., Liu, F., Yuan, J.H., Wang, F., et al. (2017). METTL14 suppresses the metastatic potential of hepatocellular carcinoma by modulating N(6) -methyladenosine-dependent primary MicroRNA processing. *Hepatology* 65(2)**,** 529-543. doi: 10.1002/hep.28885.

Mittenbühler, M.J., Saedler, K., Nolte, H., Kern, L., Zhou, J., Qian, S.B., et al. (2020). Hepatic FTO is dispensable for the regulation of metabolism but counteracts HCC development in vivo. *Mol Metab* 42**,** 101085. doi: 10.1016/j.molmet.2020.101085.

Pu, J., Wang, J., Qin, Z., Wang, A., Zhang, Y., Wu, X., et al. (2020). IGF2BP2 Promotes Liver Cancer Growth Through an m6A-FEN1-Dependent Mechanism. *Front Oncol* 10**,** 578816. doi: 10.3389/fonc.2020.578816.

Qiao, K., Liu, Y., Xu, Z., Zhang, H., Zhang, H., Zhang, C., et al. (2021). RNA m6A methylation promotes the formation of vasculogenic mimicry in hepatocellular carcinoma via Hippo pathway. *Angiogenesis* 24(1)**,** 83-96. doi: 10.1007/s10456-020-09744-8.

Qu, S., Jin, L., Huang, H., Lin, J., Gao, W., and Zeng, Z. (2021). A positive-feedback loop between HBx and ALKBH5 promotes hepatocellular carcinogenesis. *BMC Cancer* 21(1)**,** 686. doi: 10.1186/s12885-021-08449-5.

Rao, X., Lai, L., Li, X., Wang, L., Li, A., and Yang, Q. (2021). N(6) -methyladenosine modification of circular RNA circ-ARL3 facilitates Hepatitis B virus-associated hepatocellular carcinoma via sponging miR-1305. *IUBMB Life* 73(2)**,** 408-417. doi: 10.1002/iub.2438.

Rong, D., Wu, F., Lu, C., Sun, G., Shi, X., Chen, X., et al. (2021). m6A modification of circHPS5 and hepatocellular carcinoma progression through HMGA2 expression. *Mol Ther Nucleic Acids* 26**,** 637-648. doi: 10.1016/j.omtn.2021.09.001.

Shi, Y., Zhuang, Y., Zhang, J., Chen, M., and Wu, S. (2020). METTL14 Inhibits Hepatocellular Carcinoma Metastasis Through Regulating EGFR/PI3K/AKT Signaling Pathway in an m6A-Dependent Manner. *Cancer Manag Res* 12**,** 13173-13184. doi: 10.2147/cmar.S286275.

Su, R., Dong, L., Li, Y., Gao, M., He, P.C., Liu, W., et al. (2022). METTL16 exerts an m(6)A-independent function to facilitate translation and tumorigenesis. *Nat Cell Biol* 24(2)**,** 205-216. doi: 10.1038/s41556-021-00835-2.

Su, T., Huang, M., Liao, J., Lin, S., Yu, P., Yang, J., et al. (2021). Insufficient Radiofrequency Ablation Promotes Hepatocellular Carcinoma Metastasis Through N6-Methyladenosine mRNA Methylation-Dependent Mechanism. *Hepatology* 74(3)**,** 1339-1356. doi: 10.1002/hep.31766.

Sun, Z., Xue, S., Zhang, M., Xu, H., Hu, X., Chen, S., et al. (2020). Aberrant NSUN2-mediated m(5)C modification of H19 lncRNA is associated with poor differentiation of hepatocellular carcinoma. *Oncogene* 39(45)**,** 6906-6919. doi: 10.1038/s41388-020-01475-w.

Tao, L., Li, D., Mu, S., Tian, G., and Yan, G. (2022). LncRNA MAPKAPK5_AS1 facilitates cell proliferation in hepatitis B virus -related hepatocellular carcinoma. *Lab Invest*. doi: 10.1038/s41374-022-00731-9.

Tian, Q.H., Zhang, M.F., Zeng, J.S., Luo, R.G., Wen, Y., Chen, J., et al. (2019). METTL1 overexpression is correlated with poor prognosis and promotes hepatocellular carcinoma via PTEN. *J Mol Med (Berl)* 97(11)**,** 1535-1545. doi: 10.1007/s00109-019-01830-9.

Wang, M., Yang, Y., Yang, J., Yang, J., and Han, S. (2020). circ_KIAA1429 accelerates hepatocellular carcinoma advancement through the mechanism of m(6)A-YTHDF3-Zeb1. *Life Sci* 257**,** 118082. doi: 10.1016/j.lfs.2020.118082.

Wang, Y., Wang, J., Li, X., Xiong, X., Wang, J., Zhou, Z., et al. (2021). N(1)-methyladenosine methylation in tRNA drives liver tumourigenesis by regulating cholesterol metabolism. *Nat Commun* 12(1)**,** 6314. doi: 10.1038/s41467-021-26718-6.

Wu, A., Hu, Y., Xu, Y., Xu, J., Wang, X., Cai, A., et al. (2021a). Methyltransferase-Like 3-Mediated m6A Methylation of Hsa_circ_0058493 Accelerates Hepatocellular Carcinoma Progression by Binding to YTH Domain-Containing Protein 1. *Front Cell Dev Biol* 9**,** 762588. doi: 10.3389/fcell.2021.762588.

Wu, J., Pang, R., Li, M., Chen, B., Huang, J., and Zhu, Y. (2021b). m6A-Induced LncRNA MEG3 Suppresses the Proliferation, Migration and Invasion of Hepatocellular Carcinoma Cell Through miR-544b/BTG2 Signaling. *Onco Targets Ther* 14**,** 3745-3755. doi: 10.2147/ott.S289198.

Wu, S., Liu, S., Cao, Y., Chao, G., Wang, P., and Pan, H. (2022). Downregulation of ZC3H13 by miR-362-3p/miR-425-5p is associated with a poor prognosis and adverse outcomes in hepatocellular carcinoma. *Aging (Albany NY)* 14(5)**,** 2304-2319. doi: 10.18632/aging.203939.

Xia, P., Zhang, H., Xu, K., Jiang, X., Gao, M., Wang, G., et al. (2021). MYC-targeted WDR4 promotes proliferation, metastasis, and sorafenib resistance by inducing CCNB1 translation in hepatocellular carcinoma. *Cell Death Dis* 12(7)**,** 691. doi: 10.1038/s41419-021-03973-5.

Xu, H., Wang, H., Zhao, W., Fu, S., Li, Y., Ni, W., et al. (2020). SUMO1 modification of methyltransferase-like 3 promotes tumor progression via regulating Snail mRNA homeostasis in hepatocellular carcinoma. *Theranostics* 10(13)**,** 5671-5686. doi: 10.7150/thno.42539.

Xue, C., Zhao, Y., Li, G., and Li, L. (2021). Multi-Omic Analyses of the m(5)C Regulator ALYREF Reveal Its Essential Roles in Hepatocellular Carcinoma. *Front Oncol* 11**,** 633415. doi: 10.3389/fonc.2021.633415.

Yang, N., Wang, T., Li, Q., Han, F., Wang, Z., Zhu, R., et al. (2021). HBXIP drives metabolic reprogramming in hepatocellular carcinoma cells via METTL3-mediated m6A modification of HIF-1α. *J Cell Physiol* 236(5)**,** 3863-3880. doi: 10.1002/jcp.30128.

Yang, Z., Li, J., Feng, G., Gao, S., Wang, Y., Zhang, S., et al. (2017). MicroRNA-145 Modulates N(6)-Methyladenosine Levels by Targeting the 3'-Untranslated mRNA Region of the N(6)-Methyladenosine Binding YTH Domain Family 2 Protein. *J Biol Chem* 292(9)**,** 3614-3623. doi: 10.1074/jbc.M116.749689.

Yeermaike, A., Gu, P., Liu, D., and Nadire, T. (2022). LncRNA NEAT1 sponges miR-214 to promoted tumor growth in hepatocellular carcinoma. *Mamm Genome*. doi: 10.1007/s00335-022-09952-1.

Yu, J., Zhang, W., Ding, D., Hu, Y., Guo, G., Wang, J., et al. (2022). Bioinformatics Analysis Combined With Experiments Predicts PUDP as a Potential Prognostic Biomarker for Hepatocellular Carcinoma Through Its Interaction With Tumor Microenvironment. *Front Oncol* 12**,** 830174. doi: 10.3389/fonc.2022.830174.

Zhai, C.T., Tian, Y.C., Tang, Z.X., and Shao, L.J. (2021). RNA methyltransferase NSUN2 promotes growth of hepatocellular carcinoma cells by regulating fizzy-related-1 in vitro and in vivo. *Kaohsiung J Med Sci* 37(11)**,** 991-999. doi: 10.1002/kjm2.12430.

Zhang, C., Huang, S., Zhuang, H., Ruan, S., Zhou, Z., Huang, K., et al. (2020). YTHDF2 promotes the liver cancer stem cell phenotype and cancer metastasis by regulating OCT4 expression via m6A RNA methylation. *Oncogene* 39(23)**,** 4507-4518. doi: 10.1038/s41388-020-1303-7.

Zhong, L., Liao, D., Zhang, M., Zeng, C., Li, X., Zhang, R., et al. (2019). YTHDF2 suppresses cell proliferation and growth via destabilizing the EGFR mRNA in hepatocellular carcinoma. *Cancer Lett* 442**,** 252-261. doi: 10.1016/j.canlet.2018.11.006.

Zhou, T., Li, S., Xiang, D., Liu, J., Sun, W., Cui, X., et al. (2020). m6A RNA methylation-mediated HNF3γ reduction renders hepatocellular carcinoma dedifferentiation and sorafenib resistance. *Signal Transduct Target Ther* 5(1)**,** 296. doi: 10.1038/s41392-020-00299-0.

Zuo, X., Chen, Z., Gao, W., Zhang, Y., Wang, J., Wang, J., et al. (2020). M6A-mediated upregulation of LINC00958 increases lipogenesis and acts as a nanotherapeutic target in hepatocellular carcinoma. *J Hematol Oncol* 13(1)**,** 5. doi: 10.1186/s13045-019-0839-x.
